# Effects of primary seed dormancy on lifetime fitness of *Arabidopsis thaliana* in the field

**DOI:** 10.1093/aob/mcac010

**Published:** 2022-01-29

**Authors:** Froukje M Postma, Jon Ågren

**Affiliations:** Plant Ecology and Evolution, Department of Ecology and Genetics, Evolutionary Biology Centre, Uppsala University, Norbyvägen 18 D, SE-752 36 Uppsala, Sweden; Plant Ecology and Evolution, Department of Ecology and Genetics, Evolutionary Biology Centre, Uppsala University, Norbyvägen 18 D, SE-752 36 Uppsala, Sweden

**Keywords:** *Arabidopsis thaliana*, germination timing, local adaptation, natural variation, seed dormancy, structural equation modelling (SEM), temporal variation

## Abstract

**Background and Aims:**

Seed dormancy determines the environmental niche of plants in seasonal environments, and has consequences for plant performance that potentially go far beyond the seed and seedling stages. In this study, we examined the cascading effects of seed dormancy on the expression of subsequent life-history traits and fitness in the annual herb *Arabidopsis thaliana*.

**Methods:**

We planted seeds of >200 recombinant inbred lines (RILs) derived from a cross between two locally adapted populations (Italy and Sweden), and both parental genotypes at the native site of the Swedish population in three consecutive years. We quantified the relationship between primary seed dormancy and the expression of subsequent life-history traits and fitness in the RIL population with path analysis. To examine the effects of differences in dormancy on the relative fitness of the two parental genotypes, we planted dormant seeds during the seed dispersal period and non-dormant seeds during the germination period of the local population.

**Key Results:**

In the RIL population, strong primary dormancy was associated with high seedling survival, but with low adult survival and fecundity, and path analysis indicated that this could be explained by effects on germination timing, rosette size and flowering start. The relationship between primary seed dormancy and germination proportion varied among years, and this was associated with differences in seasonal changes in soil moisture. The planting of dormant and non-dormant seeds indicated that the lower primary dormancy of the local Swedish genotype contributed to its higher germination proportion in two years and to its higher fecundity in one year.

**Conclusions:**

Our results show that seed dormancy affects trait expression and fitness components across the life cycle, and suggest that among-year variation in the incidence of drought during the germination period should be considered when predicting the consequences of climatic change for population growth and evolution.

## INTRODUCTION

Early life stages can have long-lasting consequences for performance across the life cycle. Conditions early in life can influence the expression of later life-history traits, as shown in a wide range of organisms including mammals, birds, reptiles and plants (Lindström, 1999; Beckerman *et al.*, 2002; Donohue *et al.*, 2010; Donohue, 2014). Moreover, mortality rates can be high during the fragile early life stages (Kitajima and Fenner, 2000; Krebs, 2001; Poorter, 2007; Donohue *et al.*, 2010), which influences the genetic variation available for selection during later life stages.

In plants, conditions experienced during the early life stages are to a large degree set by seed dormancy. Seed dormancy is a temporary block to germination in response to germination cues in viable seeds, and determines under which conditions or season a seed can germinate (Probert, 2000; Finch-Savage and Leubner-Metzger, 2006). Dormancy is a heritable trait, and the level of seed dormancy can vary both among and within species (Baskin and Baskin, 2014).

Loci influencing dormancy have been shown to affect fitness directly by determining the proportion of seeds germinating in the field (Huang *et al.*, 2010; Postma and Ågren, 2016), and indirectly by affecting other fitness-related traits such as the timing of germination and flowering time (Huang *et al.*, 2010; Chiang *et al.*, 2012; Donohue, 2014; Postma and Ågren, 2016). By influencing the timing of germination, seed dormancy determines not only the conditions experienced by the seedling, but also the seasonal environment for the rest of the life cycle, and can thereby affect both the expression of and selection on traits expressed later in life such as plant size and flowering time (Donohue *et al.*, 2010; Donohue, 2014). Seed dormancy and the corresponding timing of germination thus have consequences that potentially go far beyond the seed and seedling stages. Strong selection on primary seed dormancy has been demonstrated in *Arabidopsis thaliana* in field experiments (Huang *et al.*, 2010; Postma and Ågren, 2016). However, the strengths of direct and indirect effects of seed dormancy on traits and fitness components across the life cycle, and how they may vary among years are not well understood.

Most plant species in temperate and artic zones, including *A. thaliana*, have physiologically dormant seeds, which means that they can increase or decrease their dormancy levels in response to environmental conditions (Finch-Savage and Leubner-Metzger, 2006; Bewley *et al.*, 2013; Baskin and Baskin, 2014). Primary dormancy is induced during seed development on the mother plant (Hilhorst, 1998; Finch-Savage and Leubner-Metzger, 2006). After dispersal, seeds can lose their primary dormancy by stratification (cold moist conditions) or by dry after-ripening (Koornneef *et al.*, 2002; Finch-Savage and Leubner-Metzger, 2006; Bewley *et al.*, 2013). The requirement of after-ripening is probably an adaptation to dry summers (Ratcliffe, 1961), when warm and dry conditions can slowly release dormancy and promote germination in autumn (Probert, 2000; Bewley *et al.*, 2013). If favourable germination conditions are not met after the loss of primary dormancy, e.g. due to light limitation, seeds can acquire secondary dormancy (Finch-Savage and Leubner-Metzger, 2006; Baskin and Baskin, 2014; Martínez-Berdeja *et al.*, 2020). As a consequence, seeds can show annual cycles of dormancy release and secondary dormancy induction.

In this study, we examined the direct and indirect effects of seed dormancy on plant fitness in the annual herb *A. thaliana*. We used an Italian and a Swedish genotype originating from native populations from close to the northern and southern margin of the native range in Europe, and recombinant inbred lines (RILs) derived from a cross between these genotypes (Ågren *et al.*, 2013). The two populations show strong local adaptation (Ågren and Schemske, 2012; Ågren *et al.*, 2013; Postma and Ågren, 2016; Ellis *et al.*, 2021). A reciprocal seed transplant experiment showed that selection during seedling establishment contributed strongly to adaptive differentiation and resulted in genetically based trade-offs (Postma and Ågren, 2016). The fitness advantage of the local genotype could be explained by differences in primary dormancy (Postma and Ågren, 2016), with the Italian genotype having higher dormancy levels than the Swedish genotype. The difference in primary dormancy corresponds to a much longer dry summer period in Italy compared to Sweden (Postma and Ågren, 2016), during which the probability of seedling mortality is high (Zacchello *et al.*, 2020).

At the Swedish site, >200 RILs and the parental genotypes were planted in three consecutive years, and the local genotype outperformed the non-local genotype in all years (Postma and Ågren, 2018). The contribution of seedling establishment to the advantage of the Swedish genotype varied in strength and direction among years. The relative fitness of RILs could be explained by the timing of germination, and there was conflicting selection on germination timing via seedling establishment vs. adult survival and fecundity in two of the three years (Postma and Ågren, 2018).

In this paper, we present new results of the 3-year experiment in Sweden. We analyse the extent to which effects of seed dormancy on seedling establishment can be explained by effects on germination proportion and seedling survival, respectively, and we examine whether effects of germination timing on rosette size and flowering start can explain associations between seed dormancy and adult survival and fecundity. We tested for effects of primary seed dormancy across the life cycle in two ways. First, we estimated the effects of genetic differences in primary dormancy and germination timing on relative fitness of the local Swedish genotype and the non-local Italian genotype by comparing the direction and strength of selection observed among primary dormant seeds planted at the time of seed dispersal, and among after-ripened non-dormant seeds planted at the time of seed germination in the local population. Second, we quantified the relationship between primary seed dormancy and the expression of life-history traits and fitness components in the RIL population with path analysis. Path analysis is a structural equation modelling (SEM) technique that tests a series of hypothesized cause–effect relationships between observed variables as a composite hypothesis (Shipley, 2000; Kline, 2011). This makes it a powerful tool to decompose the effects of life-history traits on fitness into direct and indirect effects, and reveal the underlying causal network. SEM has been used to study relationships between phenological and morphological traits and fitness components in a range of plant species (Mitchell-Olds and Bergelson, 1990; Bishop and Schemske, 1998; Albert *et al.*, 2001; Gómez *et al.*, 2006; Milla *et al.*, 2009; Latta and McCain, 2009; Fournier-Level *et al.*, 2013), and between dormancy and germination response in *A. thaliana* (Montesinos-Navarro *et al.*, 2012). However, it has not yet been used to study via which other life-history traits primary seed dormancy affects lifetime fitness.

Many life-history traits such as growth and survival are strongly influenced by seasonal changes in environmental conditions (e.g. temperature and precipitation), and among-year variation in these conditions can therefore be expected to affect both estimates of relative fitness and selection on specific traits (Siepielski *et al.*, 2009). Here, we examine how the effects of seed dormancy on life-history traits and fitness vary among years. Based on the results, we formulate a conceptual model to explain how the match between annual seed dormancy cycle and seasonal changes in soil conditions determines the relative germination proportion and seedling survival of different genotypes.

We asked the following questions: (1) What is the effect of genetic differences in primary seed dormancy on fitness and its components? (2) What are the cascading effects of seed dormancy on the expression of subsequent life-history traits (germination timing, rosette size and flowering start) and how do they influence fitness? (3) How do the direct and indirect effects of genetic variation in seed dormancy on later life-history traits and fitness vary among years, and how is this related to variation in temperature and soil moisture during the germination period?

## MATERIALS AND METHODS

### Plant material and study site

We used genotypes originating from two natural populations of *Arabidopsis thaliana* (L.) Heynh., and RILs derived from a cross between the two populations (Ågren *et al.*, 2013). The source populations are located at the northern and southern end of the species’ native distribution range in Europe: Rödåsen in north-central Sweden (62°48′N, 18°12′E) and Castelnuovo in central Italy (42°07′N, 12°29′E; Ågren and Schemske, 2012). Both populations have a winter annual life cycle, meaning that seeds germinate in autumn and flower the following spring, but the seed stage is more than twice as long in the natural population in Italy compared to Sweden (6 vs. 2 months; Postma and Ågren, 2016).

The experiments were conducted at the site of the Swedish source population. Soil temperature was recorded every hour with HOBO Temperature Data Loggers (HOBO Pro Data Logger Series H08-031-08, Onset Computer Corporation, Bourne, MA, USA), with two temperature sensors placed ~1 cm below the soil surface. We obtained hourly recordings of soil moisture measured as water potential with four sensors located ~1 cm below the soil surface (Decagon Devices, Pullman, WA, USA).

### Sowing experiments

In three consecutive years (2012–2014), we established experiments by planting primary dormant seeds of RILs and the parental genotypes during the natural seed dispersal period in early summer, and non-dormant seeds of only the parental genotypes during the natural germination period at the end of summer. The fate of the seeds was monitored from germination until the time of fruit maturation the following year, thus covering the full *A. thaliana* life cycle.

The primary dormant seeds (parental genotypes and RILs) were produced at the Swedish field site and harvested at fruit maturation. The seeds were pooled by genotype, and sown during the natural seed dispersal period (beginning of July, 14–19 d after collection). We planted 80 replicates per parental genotype of the dormant field-produced seeds in all three experiments (but due to an error only 79 replicates of the Italian genotype in the 2012 experiment), with each replicate consisting of 40 seeds sown in one cell of a plug tray. In addition, we randomly selected field-matured seeds of 219 (2012 experiment), 220 (2013 experiment) and 204 (2014 experiment) RILs to be included in the experiment, of which 164 RILs were planted in all three years. In the 2012 and 2013 experiments, we planted up to ten replicates per RIL, with each replicate consisting of 40 seeds sown in one cell of a plug tray. In the 2014 experiment, we planted five replicates per RIL, with 40 seeds per replicate for 180 RILs and between 20 and 35 seeds per replicate for 40 RILs (for more details regarding experimental procedures see Postma and Ågren, 2018).

The after-ripened, non-dormant seeds (only parental genotypes) were produced in the glasshouse, using standard glasshouse conditions (20 °C 16 h light and 16 °C 8 h dark; Postma and Ågren, 2015). The seeds had been after-ripened for 1.5 years at room temperature before being planted in the field, which ensured that both genotypes had no primary dormancy left (Postma and Ågren, 2015). In each experiment, the non-dormant glasshouse-matured seeds were planted at the end of August, during the natural germination period of the local population. Each of the two parental genotypes was planted with 80 replicates of after-ripened, non-dormant seeds in the 2012 experiment, 60 replicates in the 2013 experiment and 30 replicates in the 2014 experiment. Each replicate consisted of 40 seeds (2012 and 2013 experiments) or 20 seeds (2014 experiments) sown in one cell of a plug tray. The number of seeds sown per cell was reduced in the third year because of the high proportion of seeds germinating and the high seedling survival in the first two years.

All seeds were planted in plug trays with 160 cells each (individual cells 27 mm wide and 55 mm deep). The trays were filled with an equal mixture of local sand, gravel and unfertilized peat and sunk into the ground. Both dormant (parental genotypes and RILs) and non-dormant (parental genotypes) seeds were sown in the same randomized block design which consisted of two plug trays per block, with ten blocks in the 2012 and 2013 experiments, and five blocks in the 2014 experiment. In total, more than 230 000 seeds of the parental lines and the RILs were planted in the three experiments.

### Phenotyping

Data on germination timing, total fitness, and the fitness components seedling establishment, adult survival and fecundity in experiments planted with dormant seeds at the time of seed dispersal were analysed in Postma and Ågren (2018; all three experiments) and in Postma and Ågren (2016; only 2012 experiment), but are used in new analyses here. New data in this paper are results from the experiments planted with non-dormant seeds at the time of seed germination in the natural population, as well as data on rosette size and flowering start in the experiments planted with dormant seeds, and the decomposition of variation in seedling establishment into its two components germination proportion and seedling survival in the RIL population. Below we describe how the traits and fitness components reported in this paper were estimated.

From seed planting until the end of the germination period (mid-October, just before first snow arrival), we monitored the number of seedlings within each cell once or twice per week (12 observations) in the 2012 experiment and once or twice per month (four observations) in the 2013 and 2014 experiments. To reduce the risk that seedling turnover between censuses would go unnoticed, we noted for each seedling its developmental stage (seedling with cotyledons only or seedling with true leaves developed) and position within the cell. From these recordings, we estimated for each cell the total germination proportion (corrected for seed viability; see Postma and Ågren, 2018), mean germination timing (as day of year) and seedling survival from germination until the end of the germination period.

Only the most central seedling in each cell was monitored from seedling establishment until fruit maturation. All other seedlings were removed to prevent confounding effects of seedling density, either in October at the end of the germination period (2012 experiment) or in early spring (April) well before flowering start (2013 and 2014 experiments). In the 2013 and 2014 experiments, seeds germinated very late, and seedlings were very small in mid-October. Because, at this site, survival across the winter is negatively related to rosette size (Akiyama and Ågren, 2014), thinning in October would have markedly increased the risk that many cells would not have had a surviving plant after winter, in which case our sample size for estimating survival in spring and fecundity (and the effects of germination timing on these fitness components) would be much reduced. We therefore opted to thin only after the snow was gone in April the following year (i.e. after winter mortality had occurred). Some growth is possible under the snow that typically covers *A. thaliana* at this site from some time in October to late March – early April. However, our own observations suggest that growth under the snow is minimal, which is not surprising given the low light intensity and low temperature experienced by the plants during these months (Ågren and Schemske, 2012).

At the end of the germination period (mid-October), we estimated the rosette sizes of seedlings (12 October, 2012, 15–16 October, 2013, 17–18 October, 2014). In the 2012 experiment, we measured the widest diameter of the focal seedling in each cell directly after thinning. In the 2013 and 2014 experiments (thinning in spring), we calculated the average rosette size per cell based on measurements of the widest diameter of each seedling with true leaves and a set diameter of 3.0 mm for seedlings with cotyledons only. A very large number of seedlings with only cotyledons were observed in 2013 and 2014 (>15 000) making it impractical to measure all of them. However, subsampling indicated that diameter varied very little in this category.

We monitored flowering start for each plant (the day when the first open flower was observed) every 1–5 d during the flowering period (end of April until mid-June) in the 2013 and 2014 experiments. Flowering start was not quantified in the 2012 experiment.

At fruit maturation (end of June), we recorded adult survival (from seedling establishment at the end of the germination period in autumn until fruit maturation, correcting for the thinning of seedlings) and fecundity (number of fruits with viable seeds per surviving plant). We calculated total fitness as the number of fruits produced per planted viable seed (germination proportion × seedling survival × adult survival × fecundity), using the focal plant as a proxy for the adult survival and fecundity of removed seedlings (Postma and Ågren, 2016, 2018).

Contamination of the sowing experiment with non-experimental seeds was low: at the end of the natural germination period, the cells where seeds were planted had on average 19 times (2012 experiment), nine times (2013 experiment) and 16 times (2014 experiment) more plants than the control cells where no seeds had been planted (Postma and Ågren, 2018).

### Monitoring of the natural population

We monitored germination in the natural population of *A. thaliana* from which the Swedish genotype originates, located ~100 m from the experimental plot. In 25 squares of 10 × 10 cm, we recorded the number of seedlings, their developmental stage and their position in the square on the same days as germination was monitored in the sowing experiment.

### Relative fitness of the parental genotypes

We conducted all statistical analyses in R version 3.5.0 (R Core Team, 2018). For each experimental year, we quantified the strength of selection against the non-local, Italian parental genotype within each dormancy category (planting with primary dormant vs. non-dormant seeds) based on estimates of total fitness (number of fruits produced per viable seed planted) and its four components: (1) germination proportion (observed proportion of viable seeds that germinated), (2) seedling survival (survival of young seedlings from germination until the end of the germination period), (3) adult survival (survival from the end of the germination period until fruiting) and (4) fecundity (number of fruits produced per surviving plant).

The selection coefficient (*s*) was calculated as *s* = 1 − [mean fitness of the less fit genotype/mean fitness of the fittest genotype]. We multiplied the selection coefficient by −1 when selection favoured the non-local, Italian genotype. In this way, positive selection coefficients indicate selection against the non-local Italian genotype and negative values indicate selection against the local Swedish genotype. The difference in selection against the non-local genotype between dormant seeds planted during the natural dispersal period vs. non-dormant seeds planted during the natural germination period was calculated as *s*_*diff*_ = *s*_*dorman*t_ − *s*_*nondormant*_, where positive values indicate that genetic differences in primary dormancy increase the strength of selection favouring the Swedish genotype.

We examined the significance of the selection coefficients and of the difference in selection coefficients between the dormant and non-dormant seeds by calculating 95 % confidence intervals with the bias-corrected and accelerated method by resampling the data 10 000 times with the BOOT package in R (Canty and Ripley, 2017). We considered selection and the difference in selection, respectively, to be statistically significant when the 95 % confidence interval did not overlap zero.

### The effect of dormancy and genotype on fitness and trait expression

We used generalized linear models (GLMs) to examine the effect of genotype (Italian vs. Swedish), dormancy category (primary dormant seeds planted during the natural seed dispersal period vs. after-ripened non-dormant seeds planted during the natural germination period) and year (2012, 2013 vs. 2014 experiment) on total fitness, its components, and the traits germination timing (day of year), rosette diameter, flowering start (day of year) and number of days from germination until flowering start.

In the analyses of variation in fitness and its components, we used a negative binomial error distribution with log link function for total fitness and fecundity, a quasibinomial error distribution with logit link function for germination proportion and seedling survival, and a binomial error distribution with logit link function for adult survival. In the analyses of trait variation, we used a Poisson error distribution with log link function for germination timing, flowering start and number of days from germination until flowering start, and a quasipoisson error distribution with log link function for rosette size.

We tested the effect of genotype, dormancy category, year, and all second- and third-order interactions by comparing the change in deviance between the full model and a reduced model in which one term is dropped at a time (genotype × dormancy × year vs. same model without third-order interaction; genotype + dormancy + year + second-order interactions vs. same model with single term deletion of second-order interactions; genotype + dormancy + year vs. same model with single term deletion of genotype/dormancy/year) with a χ ^2^-test (binomial, negative binomial and Poisson error distribution) or *F*-test (quasibinomial and quasipoisson error distribution).

We tested the effect of genotype on response variables within dormancy category in each of the three experimental years with contrasts using the CONTRAST package in R (Kuhn *et al.*, 2013).

### Path analysis of RIL trait expression and fitness

We used path analysis to examine the effects of seed dormancy on three traits (germination timing, rosette size, flowering start), and the fitness components germination proportion, seedling survival, adult survival and fecundity. We used estimates of the mean dormancy levels of seeds produced at the Swedish field site in 2011, obtained for the same RILs in a previous experiment (Postma and Ågren, 2015). Seed dormancy was quantified as 1 − germination proportion after 12 weeks of after-ripening at room temperature, which reflects the after-ripening time needed for the seeds to become germinable before the onset of winter, and captures the large variation in dormancy level of field-matured seeds among RILs and among the parental genotypes (Postma and Ågren, 2015). The dormancy levels of field-matured seeds of the two parental genotypes sown in the 2012 and 2013 experiments were similar to the dormancy levels of seeds that matured at the Swedish field site in 2011 (dormancy was not quantified for the parental genotypes of the 2014 experiment; Supplementary Data Fig. S1), indicating that RIL values from the 2011 experiment are representative for RIL dormancy levels in the following field experiments.

We conducted the path analysis with the ‘lavaan’ package version 0.5-20 (Rosseel, 2012) in R. We specified our initial, hypothesized path model based on results from previous experiments conducted with seeds and seedlings planted at the Swedish field site (Akiyama and Ågren, 2014; Postma and Ågren, 2016) and other existing literature on *A. thaliana* (see Introduction). We used RIL mean values of the variables, as this reduces both environmentally induced covariances between traits and fitness (Rausher, 1992; Stinchcombe *et al.*, 2002), and influence of non-random missing observations of later life stages caused by plant mortality, which can be problematic in SEM (Beaujean, 2014). We fitted models separately for each of the three experimental years. RILs that did not have complete data in a given year were omitted from the analysis for that year (2012 experiment, five RILs; 2013 experiment, two RILs; 2014 experiment, one RIL). As we did not record flowering start in the 2012 experiment, we removed paths to and from flowering start in the model for that year.

The majority of the hypothesized paths of the model had linear relationships between variables, and none of the non-linear relationships had a clear intermediate minimum or maximum, except the relationship between dormancy and flowering date in the 2013 experiment. Including the non-linear term for the relationship between dormancy and flowering date in the 2013 experiment resulted in a model with a poor fit to the data (χ ^2^ goodness-of-fit statistic, *P* < 0.05, and the approximate fit index RMSEA > 0.05). We therefore assumed linear relationships in all models. Germination timing and flowering start were quantified as day of the year. To reduce differences in scale between variables (Rosseel, 2012), we rescaled the values of germination timing for all years, flowering start in the 2013 and 2014 experiments, and fecundity in the 2012 and 2014 experiments by dividing by a factor of 10, and fecundity in the 2013 experiment by dividing by 100.

We evaluated the fit of the model to the data using a robust maximum-likelihood estimator (MLM) with the Satorra–Bentler scaled χ ^2^ goodness-of-fit statistic and robust standard errors, as most of our variables had distributions deviating from normality (Shipley, 2000; Kline, 2011; Beaujean, 2014). In addition, we calculated the following Approximate Fit Indexes for model evaluation: comparative fit index (CFI), Tucker–Lewis index (TLI), root mean squared error (RMSEA) and standardized maximum normalized residual (SRMR). Path models are considered to fit well to the data when the χ ^2^ goodness-of-fit statistic is non-significant (*P*_M_ > 0.05), CFI and TLI > 0.95, and RMSEA and SRMR < 0.05 (Hu and Bentler, 1999; Shipley, 2000; Pugesek *et al.*, 2003). We improved our initial model by adding paths that had a modification index (MI) > 8.8, indicating that addition of the path would improve the model significantly (based on the χ ^2^ goodness-of-fit statistic), and that were biologically relevant (Pugesek *et al.*, 2003). Finally, we removed non-significant paths (*P* < 0.05) based on the Z-test (Beaujean, 2014).

## RESULTS

### Genetic differences in primary seed dormancy affect relative fitness

Comparisons of selection among the primary dormant seeds planted at the time of seed dispersal (henceforth ‘the dormant seeds’) and among the non-dormant seeds planted at the time of seed germination in the local population (‘the non-dormant seeds’) showed that genetic differences in primary dormancy affected the relative fitness of the Italian and Swedish genotypes because of effects on fitness components expressed throughout the life cycle.

**Fig. 1.**
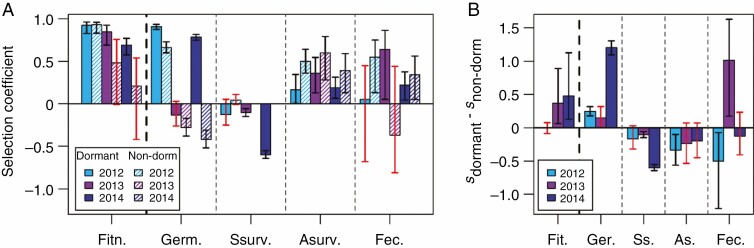
Selection coefficients (*s*) quantifying the strength of selection against the non-local Italian genotype (A) and the difference in selection against the non-local genotype when planted as dormant vs. non-dormant seeds (B) in the 2012, 2013 and 2014 experiments conducted in Sweden. Selection against the local genotype is indicated with a negative selection coefficient. Selection coefficients are based on total fitness and its components germination proportion (Germ.), seedling survival (Ssurv.), adult survival (Asurv.) and fecundity (Fec.). Bootstrapped 95 % confidence intervals that overlap zero (non-significant selection coefficients or difference in selection) are shown in red.

Among the primary dormant seeds, there was strong selection against the non-local Italian genotype in all three years (Fig. 1A; Supplementary Data Table S1). Genetic differences in dormancy between the Italian and Swedish genotype contributed to selection, favouring the Swedish genotype (Fig. 1B): the strength of selection was stronger among the dormant seeds compared to the non-dormant seeds in two of three years (2013 and 2014), and comparable in strength in one year (2012; Fig. 1).

The increased overall selection among dormant seeds favouring the local genotype was mainly the result of increased selection via fecundity (number of fruits produced per surviving plant) in the 2013 experiment, and via germination proportion (proportion of viable seeds that germinated) in the 2014 experiment (Fig. 1; Supplementary Data Table S1). Selection favouring the Swedish genotype through germination proportion was stronger among dormant than among non-dormant seeds also in the 2012 experiment, but this effect was balanced by weaker selection through adult survival (from the end of the germination period until fruiting) and fecundity (Fig. 1B).

Among plants developing from the dormant seeds, there was selection via seedling survival (survival of young seedlings from the moment of germination until the end of the germination period) favouring the Italian genotype in two of three years (Fig. 1A). Among plants developing from the non-dormant seeds, there was no significant selection through seedling survival in any of the three years (Fig. 1A; Supplementary Data Table S1).

Differences in primary dormancy affected selection through both adult survival and fecundity, but effects varied among years. Selection expressed as differences in adult survival favoured the Swedish genotype in all three years, among both dormant and non-dormant seeds (Fig. 1A; Supplementary Data Table S1). Genetic differences in primary dormancy reduced the strength of selection via adult survival significantly in one year (Fig. 1B). Selection against the Italian genotype via fecundity was stronger in one year (2013) and weaker in another year (2012) among the dormant compared with the non-dormant seeds (Fig. 1B).

Germination proportion and seedling survival were lower, whereas adult survival (mainly of the Italian genotype) and fecundity were higher among plants developing from dormant seeds compared to plants developing from non-dormant seeds (Supplementary Data Fig. S2; Table S2). Among the non-dormant seeds, the direction of the difference in germination proportion between the two genotypes varied among years (Fig. 1A; Fig. S2B). Non-dormant seeds of the Swedish genotype germinated to a higher proportion than the Italian genotype in the 2012 experiment, but the opposite was true in the 2013 and 2014 experiments.

### Genetic differences in seed dormancy have cascading effects on the expression of subsequent life-history traits

#### Trait expression of the parental genotypes.

**Fig. 2.**
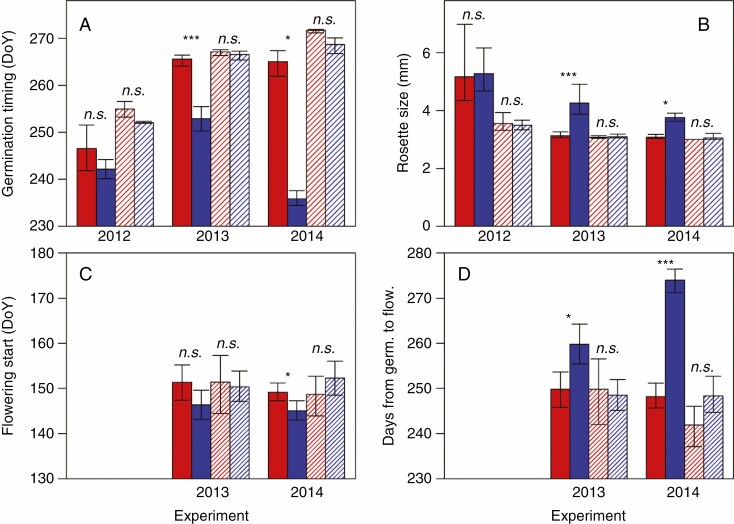
Life-history traits of the Italian (red) and Swedish (blue) genotypes planted as dormant seeds shortly after maturation at the field site (filled bars) or as non-dormant seeds at the time of seed germination in the local population (dashed bars). (A) Germination time (day of year, DoY), (B) rosette size (diameter in mm), (C) flowering start (day of year) and (D) number of days from germination until flowering start in the 2012, 2013 and 2014 experiments. Means and 95 % confidence intervals are indicated. The statistical significance of genotypic effects on life-history traits in individual years was tested with contrasts. *P < 0.05, **P < 0.01, ***P < 0.001.

Genetic variation in primary dormancy contributed to differences in germination timing, rosette diameter and flowering start between the parental genotypes (Fig. 2; Supplementary Data Tables S3 and S4). When planted as primary dormant seeds, the Swedish genotype germinated earlier and produced a larger leaf rosette than did the Italian genotype in two (2013 and, 2014) of three years (Fig. 2A, B; Table S3). In contrast, among the non-dormant seeds, the two genotypes did not differ in germination timing or rosette size in any of the three years (Fig. 2A, B).

Rosette size was related to timing of germination, but not to germination proportion (seedling density). GLMs detected significant negative relationships between germination timing and rosette size in all three years for dormant as well as non-dormant seeds (in all cases *P* ≤ 0.001), whereas estimates of the effect of germination proportion on rosette size were as often negative as positive, and statistically significant only for dormant seeds planted in 2013 when the effect was positive (*N* = 158, *P* = 0.01; Supplementary Data Table S5).

Among the dormant seeds, the Swedish genotype tended to start flowering a few days earlier than the Italian genotype (statistically significant difference in 2014; Fig. 2C). Still, because of the difference in germination time, the period from germination to flowering was markedly longer in the Swedish compared to the Italian genotype in both years (10 and 26 d longer, respectively; Fig. 2D; Supplementary Data Table S3). Among plants developing from the non-dormant seeds, there was no significant difference in flowering start between the two genotypes (Fig. 2D).

#### Path analysis of RIL mean trait and fitness values.

Path analysis indicated that primary seed dormancy affected fitness directly by affecting germination proportion and indirectly by have cascading effects on germination timing, rosette size and flowering time, but also that the strengths of effects varied among years. Fitness components and traits varied widely in the RIL population (Supplementary Data Figs S3 and S4). For all years, our final path models fitted the data well (*P*_M_ > 0.05, CFI and TLI > 0.95, and RMSEA and SRMR < 0.05; Table S6).

**Fig. 3.**
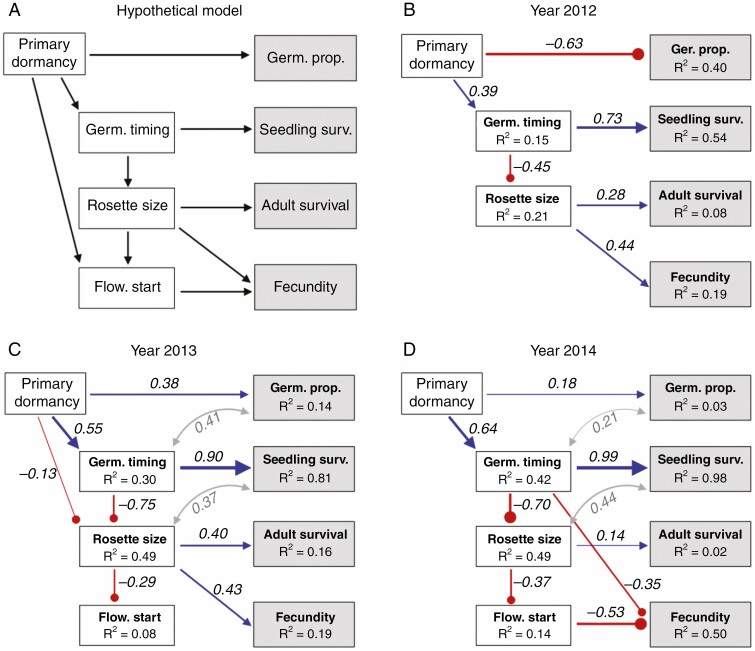
The hypothetical (A) and final models (B–D) of the path analysis based on RIL means in the 2012, 2013 and 2014 experiments. The models quantify the effect of primary seed dormancy on the traits (white boxes) germination timing (day of year), rosette size (diameter in mm) and flowering start (day of year), and the fitness components (grey boxes) germination proportion, seedling survival, adult survival and fecundity. Blue pointed arrows indicate positive effects, and red round arrows indicate negative effects. Thickness of arrows corresponds to the value of the standardized path coefficients, which are given above or beside the arrow. Flowering time was not measured in the 2012 experiment.

The path models show that the effect of seed dormancy on germination proportion varied in both strength and direction among years (Fig. 3; Supplementary Data Table S7), whereas the other relationships between traits and fitness components varied in strength and statistical significance but not in direction across the three years (Fig. 3; Table S7). The effect of seed dormancy on germination timing varied considerably among years: the unstandardized path coefficient was more than twice as large in the 2014 experiment (21.1 d) compared to the 2012 and 2013 experiments (8.4 and 10.5 d; Table S7), which is consistent with variation in the magnitude of the difference in mean germination date between the parental genotypes in the three years (Fig. 2A; Fig. S4; Table S3).

Strong seed dormancy was associated with late timing of germination in all three years, and late germination timing positively affected seedling survival: the later a seedling emerged, the higher was survival until the end of the germination period (Fig. 3; Supplementary Data Table S7). However, late germination was associated with smaller leaf rosettes, and rosette size was positively correlated with adult survival in all three years (Fig. 3; Table S7). Rosette size had a direct positive effect on fecundity in two of the three years, but affected fecundity only via flowering start in one of two years (flowering start was not measured in the 2012 experiment). Dormancy affected rosette size directly in 2013, but in no year did it have a direct effect on flowering start (Fig. 3; Table S7).

There were partial correlations among germination proportion and germination timing, and among seedling survival and rosette size in the 2013 and 2014 experiments (Fig. 3; Supplementary Data Table S7), indicating that the errors of the model are correlated. The partial correlations are probably related to variation in precision of the estimates of these variables. The total proportion of germinated seeds increased with mean germination timing in 2013 and 2014. As a result, sample sizes and precision of the estimates of germination timing, seedling survival and rosette size were higher among late-germinating RILs compared to early-germinating RILs, which may produce partial correlations of errors.

### Among-year variation in germination behaviour and seedling mortality

**Fig. 4.**
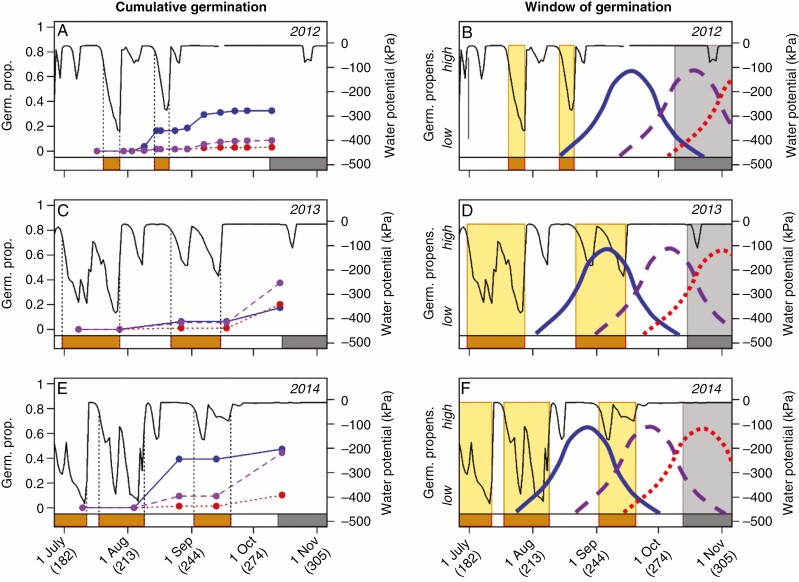
Cumulative mean germination proportion (number of seedlings per sown seed) of the Italian (red) and Swedish (blue) genotypes, and in the RIL population (purple) as a function of time (indicated as date and day of the year; A, C, E), and a conceptual model of differences in seasonal changes in germination propensity (1 − proportion of seeds that are dormant) of these three categories in relation to periods unfavourable for germination in 2012, 2013 and 2014 (B, D, F). Soil water potential at the Swedish field site during the germination period is shown in black. Dry periods (orange, soil water potential <−30 kPa for more than 7 d) and the onset of winter (grey, mean daily soil temperature below 5 °C for more than two subsequent days, see Fig. 5) should constitute conditions unfavourable for germination and are indicated on the *x*-axes.

The effects of genotype on the proportion of seeds germinating, germination timing and seedling survival until winter varied among years (Figs 2A, 4A, C, E; Supplementary Data Fig. S2C), and this variation was associated with differences in the timing and duration of dry periods during the germination season. Between seed dispersal and onset of winter, each experiment had two to three periods with low soil moisture lasting for more than 7 d (orange blocks on *x*-axes in Fig. 4). During these periods very few seeds germinated (Fig. 4A, C, E), and seedling mortality was high (Fig. S5).

Among dormant seeds, the Swedish genotype reached a much higher total proportion of germination compared with the Italian genotype in the 2012 and 2014 experiments, while no significant difference between the two genotypes was observed in the 2013 experiment (Fig. 4A, C, E; Supplementary Data Fig. S2B). Interestingly, by the end of the germination period in the 2013 experiment, the mean proportion of seeds that had germinated in the RIL population was higher than that of the two parental genotypes. The Italian and Swedish genotypes differ in the timing of their annual seed dormancy cycles (Postma *et al.*, 2016; Zacchello, 2021), and the among-year variation in germination proportion of different genotypes may reflect differences in the match between the timing of dormancy release and seasonal changes in conditions for germination, as suggested by the conceptual model illustrated in Fig. 4B, D and F.

Among-year variation in the timing and duration of periods with low soil moisture was associated also with variation in the magnitude of differences between the Italian and Swedish genotype with regard to germination timing and seedling survival. In 2014, almost all seeds of the Swedish genotype germinated before the more than 2-week-long period of drought in early September, whereas the majority of Italian seeds germinated only after this period, and differences in germination timing (~4 weeks; Figs 2A, 4E) and seedling survival until winter (Italian genotype 90 %, Swedish genotype 36 %; Supplementary Data Fig. S2C; Table S1) were particularly large. In 2013, an even longer dry period began as early as late August. In this year, a rather small proportion of the Swedish seeds had germinated before the onset of drought, germination of both genotypes peaked after the dry period, and the differences in germination timing (~2 weeks; Figs 2A, 4C) and seedling survival (Italian genotype 97 %, Swedish genotype 87 %; Fig. S2C; Table S1) were smaller. Finally, in 2012 the proportion of Italian seeds that germinated was very low, the difference in timing of germination was small and not statistically significant (Figs 2A, 4A), and seedling survival of the Italian genotype was somewhat higher than that of the Swedish genotype (71 % vs. 62 %; Fig. S2C; Table S1).

The local Swedish genotype had the same seasonal pattern of germination as the natural population of *A. thaliana* from which the Swedish genotype originates, located about 100 m from the experiment (Supplementary Data Fig. S5). However, in 2012, seedling survival in the natural population was apparently not as strongly affected by the drought in August as plants in the experiment, but was markedly reduced during the last month before winter (Fig. S5).

## DISCUSSION

The results of (1) the comparison between the Swedish and Italian genotypes planted as primary dormant seeds and as after-ripened non-dormant seeds, and (2) the path model based on seed fates in the RIL population show that loci affecting seed dormancy influence several life-history traits and fitness components across the whole life cycle. Genetic differences in primary dormancy contributed to the fitness advantage of the local genotype, and the path models showed that genetic variation in primary dormancy affected fitness components via germination timing, rosette size and flowering start. Moreover, data on seasonal changes in soil moisture and temperature suggest that differences in soil conditions during the germination period contribute to among-year variation in the strength and direction of selection during the germination phase. Below, we discuss our results in relation to previous studies on the cascading effects of seed dormancy, and the effect of environmental conditions on dormancy and germination behaviour.

### Dormancy contributes to genotypic differences in fitness and life-history traits

In previous studies with the same study system, we have shown that selection during the seedling establishment phase contributes strongly to adaptive differentiation (Postma and Ågren, 2016), but that its contribution can vary markedly among years (Postma and Ågren, 2018). By decomposing effects on seedling establishment into those on germination proportion and seedling survival, the present study shows that the genetic difference in primary dormancy contributed to selection favouring the local Swedish genotype by affecting relative germination proportion in two years and relative fecundity in one year. It also revealed that the Italian genotype had higher seedling survival in autumn compared with the Swedish genotype, which is consistent with a positive correlation between seedling survival until winter and germination timing documented in a previous experiment at this site manipulating germination timing of the local genotype (Akiyama and Ågren, 2014). By germinating late, the Italian genotype to a large extent escaped the periods of drought recorded in the first half of September in 2013 and 2014. In addition, the late germination was associated with a very short period of growth and exposure to other possible mortality factors before the onset of winter and the appearance of snow cover.

Differences in primary dormancy contributed to differences in germination timing and rosette size between the Italian and Swedish genotype. Path analysis indicated that primary dormancy was positively associated with germination timing, which in turn was negatively related to rosette size, and these relationships were consistent in the RIL population across the three years of study (Fig. 3). Moreover, when differences in primary dormancy were allowed to be expressed, the non-local Italian genotype germinated later and produced a smaller leaf rosette compared to the local Swedish genotype in two of the three years. Late germination is expected to be associated with small leaf rosettes at this site both because the time available for growth before the onset of winter becomes short, and because conditions for growth deteriorate as air temperature and the period of daylight successively decrease during autumn (Akiyama and Ågren, 2014). Finally, the Swedish genotype planted as primary dormant seeds had the same seasonal pattern of germination as the natural population of *A. thaliana* from which it originates (Supplementary Data Fig. S5), demonstrating that the planting of dormant seeds at the time of seed dispersal mimics the natural life cycle well.

Among plants developing from seeds planted in a dormant state at the time of seed dispersal, the Italian genotype tended to start flowering a few days later than the Swedish genotype (statistically significant difference in the 2014 experiment; Fig. 2C). In contrast, in previous field experiments at the same site in which seedlings were planted, the Italian genotype began flowering about 3–9 d earlier than the Swedish genotype (Ågren and Schemske, 2012; Ågren *et al.*, 2017). In addition, in a glasshouse experiment with simulated Swedish climate where differences in seed dormancy were reduced by stratification before sowing, the Italian genotype began flowering 10 d earlier than the Swedish genotype (Dittmar *et al.*, 2014). This illustrates the importance of including the early life stages in experiments assessing causes of variation in flowering time in the field.

When planted as dormant seeds, the difference in flowering start between the Italian and Swedish genotype was rather small, but because of their different germination times, the two genotypes differed markedly in number of days from germination to flowering (Fig. 2D). The late germination of the Italian genotype compared to the Swedish genotype (13 d later in the 2013 experiment, and 29 d later in the 2014 experiment) was compensated for by a shorter pre-flowering period, resulting in a flowering start rather similar to that of the Swedish genotype.

### Dormancy has cascading effects across the life cycle

Analysis of the relationships between primary seed dormancy, life-history traits and fitness components in the RIL population demonstrated that seed dormancy influenced all fitness components, directly by influencing the proportion of seeds germinating, and indirectly via effects on the timing of germination, rosette size and flowering start. Consistent with the results of the present study, seed dormancy was found to affect germination timing and germination proportion of *A. thaliana* in a field experiment conducted in the introduced range in North America (Huang *et al.*, 2010). Both studies thus support the prediction that seed dormancy has cascading effects on major life-history traits across the life cycle, thereby affecting overall fitness in *A. thaliana* (cf. Donohue *et al.*, 2010; Donohue, 2014).

The present results provide further insight into selection on primary seed dormancy at the study site and how it differs from that at the native site of the Italian genotype. In the present study, strong dormancy and late germination were correlated with high seedling survival before winter. However, late germination provided little time and poor conditions for growth before the onset of winter, resulting in small leaf rosettes, and was associated with low adult survival and fecundity. Similarly, a previous field experiment in which the timing of germination was manipulated indicated that germination time was subject to conflicting selection at this site: late germination was associated with increased seedling survival until the onset of winter, but this was more than balanced by reduced adult survival and fecundity (Akiyama and Ågren, 2014). In that experiment, the highest fitness was achieved by plants germinating in August, about 1.5 months after seed maturation. By contrast, at the native site of the Italian genotype, experimental manipulation of germination time indicated strong stabilizing selection with an optimal germination time in November, ~6 months after seed maturation, with no evidence of conflicting selection through seedling survival vs. adult survival and fecundity (Zacchello *et al.*, 2020). No successful seedling establishment was recorded before November, and all components of fitness were reduced when germination occurred later than November. Differences in the length of the dry summer period and optimal timing of germination are thus likely to contribute to the maintenance of the difference in seed dormancy between the Italian and Swedish genotype (Postma and Ågren, 2016).

Seed dormancy influenced flowering time indirectly via rosette size, but path analysis did not detect any direct effect of differences in seed dormancy on flowering time (Fig. 3C, D). The Italian and Swedish genotypes differ with regard to haplotype at the seed dormancy locus DELAY OF GERMINATION 1 (DOG1), and quantitative trait locus (QTL) mapping has indicated that differences in the region of this locus can explain much of the difference in seed dormancy between the two genotypes (Postma and Ågren, 2015). Pleiotropic effects of DOG1 on flowering time independent of its effects on germination timing have been detected (Atwell *et al.*, 2010; Huo *et al.*, 2016; Martínez-Berdeja *et al.*, 2020). However, when non-dormant seeds were planted during the germination period of the local population, the Italian and Swedish genotypes did not differ in flowering time (Fig. 2C, D). This suggests that these two haplotypes of DOG1 do not have a pleiotropic effect on flowering time independent of differences in germination timing in this environment, or that any such difference is balanced by differences at other loci affecting flowering time. Near-isogenic lines could be used to distinguish between these two alternatives.

### Among-year variation in germination can be explained by variation in soil conditions

The effects of genetic differences in seed dormancy on fitness showed particularly high among-year variation during the seed germination stage. First, the effect of primary dormancy on germination proportion in the RIL population varied not only in strength but also in direction among the three experimental years (Fig. 3; Supplementary Data Table S7). Second, both comparisons of the two parental genotypes (Fig. 2A) and relationships in the RIL population (Table S7) showed that the effect of genetic differences in primary dormancy on timing of germination varied greatly in strength among years.

Among-year variation in the match between annual seed dormancy cycle and seasonal changes in soil conditions could potentially explain why the relative germination success and seedling survival of the two genotypes varied among years (illustrated with a conceptual model in Fig. 4B, D, F). Previous work at the study site has shown that the Italian and Swedish genotypes differ in their annual seed dormancy cycle, with the Swedish genotype releasing its seed dormancy earlier compared to the Italian genotype (Postma *et al.*, 2016; Zacchello, 2021), and that this is associated with earlier germination (Postma and Ågren, 2018; see also Fig. 2A). Less is known about the relative timing of secondary dormancy induction. In our conceptual model, we portray a situation where the period between release of seed dormancy and renewed secondary dormancy is equally long in the two parental genotypes and in the RIL population. Germination cannot occur during periods with low soil moisture (Fig. 4, orange blocks), or towards the end of autumn when soil temperatures become too low (Fig. 4, grey blocks). With these assumptions, the match between the annual seed dormancy cycle and seasonal changes in soil moisture and temperature will determine the proportion of seeds that will germinate, and among-year differences in this match could help explain variation in cumulative germination curves of the two parental genotypes and the RIL population.

**Fig. 5.**
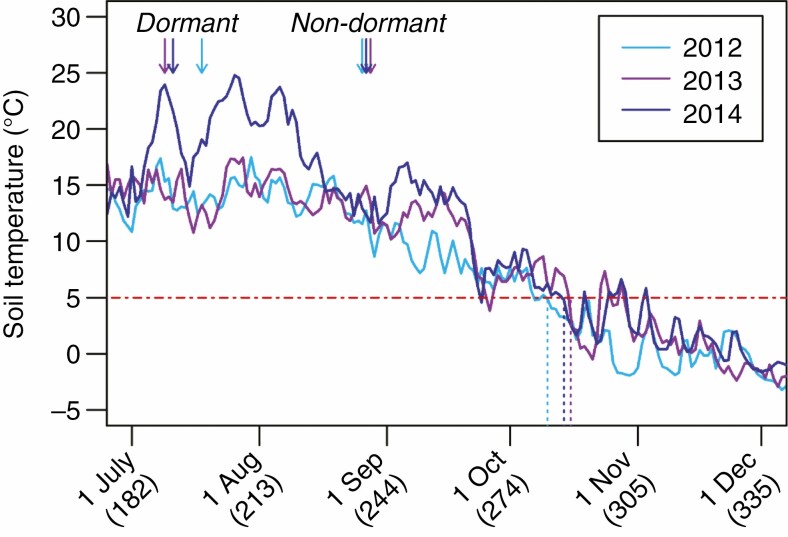
Mean daily soil temperature at the experimental site in Sweden in the 2012, 2013 and 2014 experiments. The times of planting of dormant and non-dormant seeds are indicated with arrows. The end of the germination period, defined as the day when the mean daily soil temperature drops below 5 °C (red dashed line) for more than two subsequent days, is indicated for each experimental year with a dashed line. Date (day of year) is indicated on the *x*-axis.

For a given year and genotype, the seasonal pattern of germination will depend on the timing of seed dormancy release and during which periods conditions are conducive for germination. Temperature affects the rate of seed dormancy loss, while drought may prevent the germination of non-dormant seeds. High temperatures during seed after-ripening decrease the primary dormancy level of dormant seeds (Footitt *et al.*, 2011; Baskin and Baskin, 2014). Soil temperature in July and August was much higher in 2014 compared to the previous two years, and soil temperature in September was much higher in 2013 and 2014 compared to 2012 (Fig. 5). This may have resulted in an earlier loss of seed dormancy (Fig. 4B, D, F), giving the highly dormant Italian genotype and the RIL population with intermediate dormancy levels the possibility to reach higher germination proportions in the last two years compared to the first year (Fig. 4A, C, E). The magnitudes of differences in germination timing and seedling survival between the Italian and Swedish genotypes varied among years and were associated with the incidence of drought periods relative to the onset of germination. In 2014, most Swedish seeds germinated before a more than 2-week-long period of drought in early September, whereas the great majority of Italian seeds germinated only after this period. In this year, the mean time of germination differed by as much as 4 weeks, and the survival until the onset of winter was 2.5 times higher for the Italian compared to the Swedish genotype. In 2013, most seeds of both genotypes germinated after the drought period that lasted from late August to mid-September, and the differences in timing of germination and seedling survival were smaller (Figs 2A, 4). To identify the drivers of the among-year variation in germination patterns and selection on seed dormancy, we suggest that seasonal patterns of soil moisture and temperature should be manipulated in future field experiments.

### After-ripened non-dormant seeds can develop secondary dormancy

After primary dormancy has been released by after-ripening, seeds can subsequently acquire secondary dormancy preventing response to germination cues (Hilhorst, 1998; Finch-Savage and Leubner-Metzger, 2006; Baskin and Baskin, 2014; Martínez-Berdeja *et al.*, 2020). In no year did the dormant or non-dormant seeds planted at the field site reach 100 % germination (Supplementary Data Fig. S2B). Although a few seeds could have germinated and died unobserved, it is likely that most seeds that did not germinate acquired secondary dormancy and/or were washed deeper into the soil where they did not receive the necessary germination cues.

Among the non-dormant seeds planted at the time of seed germination, the direction of the difference in germination proportion between the Swedish and Italian genotype varied among years. In the 2012 experiment, the germination proportion of the Swedish genotype was more than twice as high as that of the Italian genotype, whereas in the 2013 and 2014 experiments the germination proportion of the Italian genotype was higher than that of the Swedish genotype (Supplementary Data Fig. S2B). This strong genotype × year interaction for germination proportion could reflect a difference between the two genotypes in the rate at which secondary dormancy is acquired in the autumn, and thereby the duration of the period when germination can be triggered. Secondary dormancy can be induced in seeds of *A. thaliana* by low water potential, and by wet incubation at both low and high temperatures (Penfield and Springthorpe, 2012; Auge *et al.*, 2015; Edwards *et al.*, 2016; Coughlan *et al.*, 2017). The lower water availability and higher soil temperature in the weeks after the planting of the non-dormant seeds in 2013 and 2014 compared to 2012 (Figs 4, 5) appear to have induced secondary dormancy to a greater extent in the Swedish genotype compared to the Italian genotype.

We have not included potential recruitment from the seed bank in our fitness estimates, but the secondary dormancy cycles observed in previous seed burial experiments at the field site suggest that potential recruitment from the seed bank would increase the advantage of the local genotype (Postma *et al.*, 2016; Zacchello, 2021).

## CONCLUSIONS

In this study genetic differences in primary seed dormancy influenced plant fitness both directly by affecting the proportion of seeds germinating and indirectly by having cascading effects on other life-history traits, influencing plant survival and fecundity. We conclude that it is vital to include the very early life stages in field experiments to gain a full understanding of the evolution of life-history traits and local adaptation. This is true, not least when assessing the effects of climatic change on population growth and evolution (Parmesan and Hanley, 2015). In the present study, differences in the incidence and duration of drought during the germination period were associated with among-year variation in the effects of seed dormancy on germination proportion, germination phenology and seedling survival. The results suggest that the effects of climate change on temperature and soil moisture during the germination season will be important to consider when predicting its consequences for population fitness and selection regimes.

## SUPPLEMENTARY DATA

Supplementary data are available online at https://academic.oup.com/aob and consist of the following. Fig. S1: germination proportion of seeds of the Italian and Swedish genotypes produced at the field site as a function of seed after-ripening. Fig. S2: fitness and fitness components of the Italian and Swedish genotypes when planted as dormant seeds shortly after maturation at the field site and as non-dormant seeds during the germination period of the local population. Fig. S3: frequency distributions of mean proportion of viable seeds germinating and mean seedling survival in the RIL population. Fig. S4: frequency distributions of mean timing of germination, rosette size, flowering start and number of days from germination until flowering start in the RIL population. Fig. S5: mean number of living seedlings expressed as a percentage of total germination of the Italian genotype, the Swedish genotype and the RIL population planted as primary dormant seeds shortly after maturation at the field site. Table S1: fitness and fitness components of the Italian and Swedish genotypes planted as primary dormant and as after-ripened non-dormant seeds, and of the RIL population planted as primary dormant seeds. Table S2: effects of year, genotype and dormancy category on total fitness and its components. Table S3: life-history traits of the Italian and Swedish genotypes planted as primary dormant seeds, and as after-ripened non-dormant seeds, and of the RIL population planted as primary dormant seeds. Table S4: effects of year, genotype and dormancy category on life-history traits. Table S5: effects of germination timing and germination proportion on rosette size, analysed separately by year and dormancy category. Table S6: sequential model evaluation in the path analysis of effects of primary dormancy on life-history traits and fitness in the RIL population. Table S7: unstandardized path coefficients in the final models of the path analysis for the 2012, 2013 and 2014 experiments.

mcac010_suppl_Supplementary_Material

## References

[CIT0001] Ågren J , Oakley CG, Lundemo S, Schemske DW. 2017. Adaptive divergence in flowering time among natural populations of *Arabidopsis thaliana*: Estimates of selection and QTL mapping. Evolution 71: 550–564. 27859214 10.1111/evo.13126

[CIT0002] Ågren J , Oakley CG, McKay JK, Lovell JT, Schemske DW. 2013. Genetic mapping of adaptation reveals fitness tradeoffs in *Arabidopsis thaliana*. Proceedings of the National Academy of Sciences 110: 21077–21082. 10.1073/pnas.1316773110 PMC3876199 24324156

[CIT0003] Ågren J , Schemske DW. 2012. Reciprocal transplants demonstrate strong adaptive differentiation of the model organism *Arabidopsis thaliana* in its native range. New Phytologist 194: 1112–1122. 22432639 10.1111/j.1469-8137.2012.04112.x

[CIT0004] Akiyama R , Ågren J. 2014. Conflicting selection on the timing of germination in a natural population of *Arabidopsis thaliana*. Journal of Evolutionary Biology 27: 193–199. 24329869 10.1111/jeb.12293

[CIT0005] Albert MJ , Escudero A, Iriondo JM. 2001. Female reproductive success of narrow endemic *Erodium paularense* in contrasting microhabitats. Ecology 82: 1734–1747.

[CIT0006] Atwell S , Huang YS, Vilhjálmsson BJ, et al 2010. Genome-wide association study of 107 phenotypes in *Arabidopsis thaliana* inbred lines. Nature 465: 627–631. 20336072 10.1038/nature08800 PMC3023908

[CIT0007] Auge GA , Blair LK, Burghardt LT, et al 2015. Secondary dormancy dynamics depends on primary dormancy status in *Arabidopsis thaliana*. Seed Science Research 25: 230–246.

[CIT0008] Baskin CC , Baskin JM. 2014. Seeds: ecology, biogeography, and evolution of dormancy and germination. San Diego: Elsevier.

[CIT0009] Beaujean AA . 2014. Latent Variable Modeling using R: a step-by-step guide. New York: Routledge.

[CIT0010] Beckerman A , Benton TG, Ranta E, Kaitala V, Lundberg P. 2002. Population dynamic consequences of delayed life-history effects. Trends in Ecology and Evolution 17: 263–269.

[CIT0011] Bewley JD , Bradford KJ, Hilhorst HWM, Nonogaki H. 2013. Seeds: physiology of development, germination and dormancy. New York: Springer.

[CIT0012] Bishop JG , Schemske DW. 1998. Variation in flowering phenology and its consequences for lupines colonizing mount St. Helens. Ecology 79: 534–546.

[CIT0013] Canty A , Ripley B. 2017. boot: bootstrap R (S-Plus) functions. R package version 1.3-20. https://CRAN.R-project.org/package=boot

[CIT0014] Chiang GCK , Barua D, Dittmar E, et al 2012. Pleiotropy in the wild: the dormancy gene *DOG1* exerts cascading control on life cycles. Evolution 67: 883–893. 23461337 10.1111/j.1558-5646.2012.01828.x

[CIT0015] Coughlan JM , Saha A, Donohue K. 2017. Effects of pre- and post-dispersal temperature on primary and secondary dormancy dynamics in contrasting genotypes of *Arabidopsis thaliana* (Brassicaceae). Plant Species Biology 32: 210–222.

[CIT0016] Dittmar EL , Oakley CG, Ågren J, Schemske DW. 2014. Flowering time QTL in natural populations of *Arabidopsis thaliana* and implications for their adaptive value. Molecular Ecology 23: 4291–4303. 25039363 10.1111/mec.12857

[CIT0017] Donohue K . 2014. Why ontogeny matters during adaptation: developmental niche construction and pleiotropy across the life cycle in *Arabidopsis thaliana*. Evolution 68: 32–47. 24117399 10.1111/evo.12284

[CIT0018] Donohue K , Rubio de Casas R, Burghardt L, Kovach K, Willis CG. 2010. Germination, postgermination adaptation, and species ecological ranges. Annual Review of Ecology, Evolution, and Systematics 41: 293–319.

[CIT0019] Edwards BR , Burghardt LT, Zapata-Garcia M, Donohue K. 2016. Maternal temperature effects on dormancy influence germination responses to water availability in *Arabidopsis thaliana*. Environmental and Experimental Botany 126: 55–67.

[CIT0020] Ellis TJ , Postma FM, Oakley CG, Ågren J. 2021. Life-history trade-offs and the genetic basis of fitness in *Arabidopsis thaliana*. Molecular Ecology 30: 2846–2858. 33938082 10.1111/mec.15941

[CIT0021] Finch-Savage WE , Leubner-Metzger G. 2006. Seed dormancy and the control of germination. New Phytologist 171: 501–523. 16866955 10.1111/j.1469-8137.2006.01787.x

[CIT0022] Footitt S , Douterelo-Soler I, Clay H, Finch-Savage WE. 2011. Dormancy cycling in *Arabidopsis* seeds is controlled by seasonally distinct hormone-signaling pathways. Proceedings of the National Academy of Sciences 108: 20236–20241. 10.1073/pnas.1116325108 PMC3250134 22128331

[CIT0023] Fournier-Level A , Wilczek AM, Cooper MD, et al 2013. Paths to selection on life history loci in different natural environments across the native range of *Arabidopsis thaliana*. Molecular Ecology 22: 3552–3566. 23506537 10.1111/mec.12285

[CIT0024] Gómez JM , Perfectti F, Camacho JPM. 2006. Natural selection on *Erysimum mediohispanicum* flower shape: insights into the evolution of zygomorphy. The American Naturalist 168: 531–545. 10.1086/507048 17004224

[CIT0025] Hilhorst HW . 1998. The regulation of secondary dormancy. The membrane hypothesis revisited. Seed Science Research 8: 77–90.

[CIT0026] Hu L , Bentler PM. 1999. Cutoff criteria for fit indexes in covariance structure analysis: Conventional criteria versus new alternatives. Structural Equation Modeling: A Multidisciplinary Journal 6: 1–55.

[CIT0027] Huang X , Schmitt J, Dorn L, et al 2010. The earliest stages of adaptation in an experimental plant population: strong selection on QTLs for seed dormancy. Molecular Ecology 19: 1335–1351. 20149097 10.1111/j.1365-294X.2010.04557.x

[CIT0028] Huo H , Wei S, Bradford KJ. 2016. *DELAY OF GERMINATION1* (*DOG1*) regulates both seed dormancy and flowering time through microRNA pathways. Proceedings of the National Academy of Sciences 113: E2199–E2206. 10.1073/pnas.1600558113 PMC4839450 27035986

[CIT0029] Kitajima K , Fenner M. 2000. Ecology of seedling regeneration. In: Fenner M, ed. Seeds: the ecology of regeneration in plant communities. Wallingford: CABI Publishing, 331–359.

[CIT0030] Kline RB . 2011. Principles and practice of structural equation modeling. New York: The Guilford Press.

[CIT0031] Koornneef M , Bentsink L, Hilhorst H. 2002. Seed dormancy and germination. Current Opinion in Plant Biology 5: 33–36. 11788305 10.1016/s1369-5266(01)00219-9

[CIT0032] Krebs CJ . 2001. Ecology. San Francisco: Benjamin Cummings.

[CIT0033] Kuhn M , Weston S, Wing J, James JF, Thaler T. 2013. Contrast: a collection of contrast methods. R package version 0.19. https://CRAN.R-project.org/package=contrast

[CIT0034] Latta RG , McCain C. 2009. Path analysis of natural selection via survival and fecundity across contrasting environments in *Avena barbata*. Journal of Evolutionary Biology 22: 2458–2469. 19824926 10.1111/j.1420-9101.2009.01857.x

[CIT0035] Lindström J . 1999. Early development and fitness in birds and mammals. Trends in Ecology and Evolution 14: 343–348. 10441307 10.1016/s0169-5347(99)01639-0

[CIT0036] Martínez-Berdeja A , Stitzer MC, Taylor MA, et al 2020. Functional variants of *DOG1* control seed chilling responses and variation in seasonal life-history strategies in *Arabidopsis thaliana*. Proceedings of the National Academy of Sciences 117: 2526–2534. 10.1073/pnas.1912451117 PMC7007534 31964817

[CIT0037] Milla R , Escudero A, Iriondo JM. 2009. Inherited variability in multiple traits determines fitness in populations of an annual legume from contrasting latitudinal origins. Annals of Botany 103: 1279–1289. 19318383 10.1093/aob/mcp068 PMC2685322

[CIT0038] Mitchell-Olds T , Bergelson J. 1990. Statistical genetics of an annual plant, *Impatiens capensis*. 11. Natural selection. Genetics 124: 417–421. PMC1203933 2307362

[CIT0039] Montesinos-Navarro A , Picó FX, Tonsor SJ. 2012. Clinal variation in seed traits influencing life cycle timing in *Arabidopsis thaliana*. Evolution 66: 3417–3431. 23106707 10.1111/j.1558-5646.2012.01689.x

[CIT0040] Parmesan C , Hanley ME. 2015. Plants and climate change: complexities and surprises. Annals of Botany 116: 849–864. 26555281 10.1093/aob/mcv169 PMC4640131

[CIT0041] Penfield S , Springthorpe V. 2012. Understanding chilling responses in *Arabidopsis* seeds and their contribution to life history. Philosophical Transactions of the Royal Society B: Biological Sciences 367: 291–297. 10.1098/rstb.2011.0186 PMC3223808 22144391

[CIT0042] Poorter L . 2007. Are species adapted to their regeneration niche, adult niche, or both? The American Naturalist 169: 433–442. 10.1086/512045 17427120

[CIT0043] Postma FM , Ågren J. 2015. Maternal environment affects the genetic basis of seed dormancy in *Arabidopsis thaliana*. Molecular Ecology 24: 785–797. 25640699 10.1111/mec.13061

[CIT0044] Postma FM , Ågren J. 2016. Early life stages contribute strongly to local adaptation in *Arabidopsis thaliana*. Proceedings of the National Academy of Sciences 113: 7590–7595. 10.1073/pnas.1606303113 PMC4941467 27330113

[CIT0045] Postma FM , Ågren J. 2018. Among-year variation in selection during early life stages and the genetic basis of fitness in *Arabidopsis thaliana*. Molecular Ecology 27: 2498–2511. 29676059 10.1111/mec.14697

[CIT0046] Postma FM , Lundemo S, Ågren J. 2016. Seed dormancy cycling and mortality differ between two locally adapted populations of *Arabidopsis thaliana*. Annals of Botany 117: 249–256. 26637384 10.1093/aob/mcv171 PMC4724045

[CIT0047] Probert RJ . 2000. The role of temperature in the regulation of seed dormancy and germination. In: Fenner M, ed. Seed: the ecology of regeneration in plant communities. Wallingford: CAB International, 261–292.

[CIT0048] Pugesek BH , Tomer A, von Eye A. 2003. Structural equation modeling: applications in ecological and evolutionary biology. Cambridge: Cambridge University Press.

[CIT0049] R Core Team. 2018. R: a language and environment for statistical computing. Vienna: R Foundation for Statistical Computing.

[CIT0050] Ratcliffe D . 1961. Adaptation to habitat in a group of annual plants. The Journal of Ecology 49: 187.

[CIT0051] Rausher MD . 1992. The measurement of selection on quantitative traits – biases due to environmental covariances between traits and fitness. Evolution 46: 616–626. 28568666 10.1111/j.1558-5646.1992.tb02070.x

[CIT0052] Rosseel Y . 2012. Lavaan: An R package for structural equation modeling. Journal of Statistical Software 48: 1–36.

[CIT0053] Shipley B . 2000. Cause and correlation in biology: a user’s guide to path analysis, structural equations and causal inference. Cambridge: Cambridge University Press.

[CIT0054] Siepielski AM , DiBattista JD, Carlson SM. 2009. It’s about time: the temporal dynamics of phenotypic selection in the wild. Ecology Letters 12: 1261–1276. 19740111 10.1111/j.1461-0248.2009.01381.x

[CIT0055] Stinchcombe JR , Rutter MT, Burdick DS, Tiffin P, Rausher MD, Mauricio R. 2002. Testing for environmentally induced bias in phenotypic estimates of natural selection: theory and practice. The American Naturalist 160: 511–523. 10.1086/342069 18707526

[CIT0056] Zacchello G . 2021. *Ecology and evolution of local adaptation in* Arabidopsis thaliana. PhD thesis, Uppsala University, Sweden.

[CIT0057] Zacchello G , Vinyeta M, Ågren J. 2020. Strong stabilizing selection on timing of germination in a Mediterranean population of *Arabidopsis thaliana*. *American Journal of Botany* 107: 1518–1526. 33058187 10.1002/ajb2.1549 PMC7756891

