# Supplementary material for: Effects of primary seed dormancy on lifetime fitness of Arabidopsis thaliana in the field
Source: Ann Bot. 2022 Jan 29;129(7):795–808. doi: 10.1093/aob/mcac010 (PMC9292592; doi:10.1093/aob/mcac010)
Supplement: mcac010_suppl_Supplementary_Material [file mcac010_suppl_supplementary_material.pdf]

Supporting information

Effects of primary seed dormancy on life-time fitness of *Arabidopsis thaliana* in the field

Froukje M. Postma and Jon Ågren

This file includes Figs. S1-S5, and Tables S1-S7.

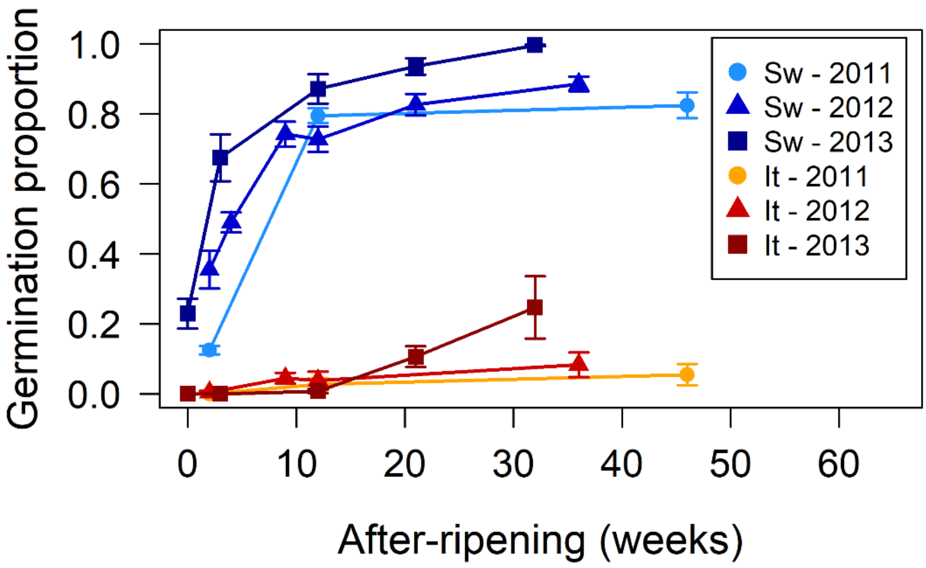

Fig. S1. The mean ± SE germination proportion as a function of seed after-ripening. Seeds of the Italian (red, filled symbols) and Swedish genotype (blue, open symbols) were harvested at the Swedish field site in June in the year 2011 (circles), 2012 (triangles) and 2013 (squares). The germination proportions were obtained from germination assays performed in a growth room under standard conditions (20 °C 16 h light and 16 °C 8 h dark) during 7 days. All seeds were fully viable.

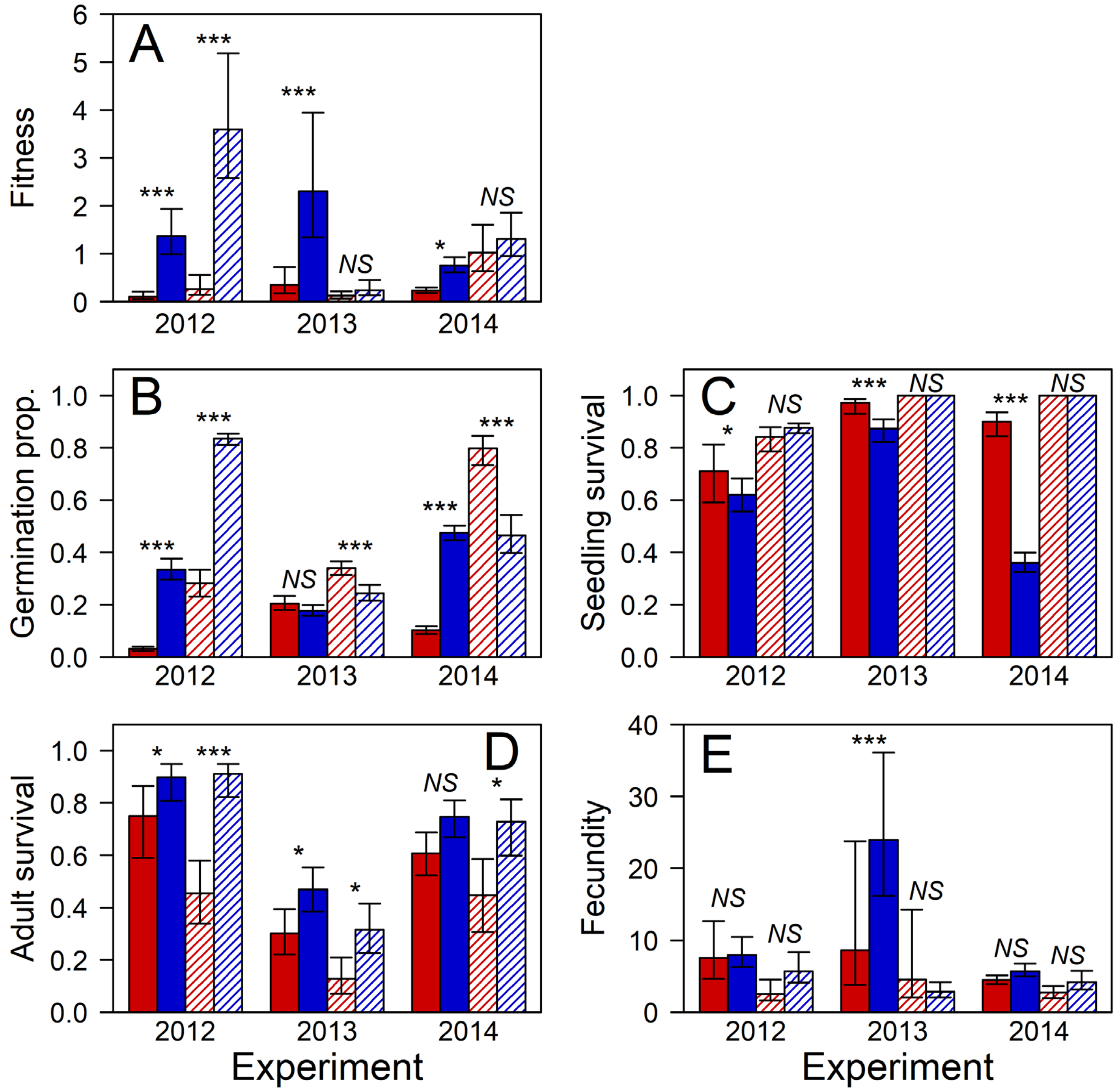

Fig. S2. Fitness and fitness components of the Italian (red) and Swedish (blue) genotypes when planted as dormant seeds shortly after maturation at the field site (filled bars) and as non-dormant seeds during the germination period of the local population (dashed bars). Total fitness (A) and its components germination proportion (B), seedling survival (C), adult survival (D), and fecundity (E) in the 2012, 2013 and 2014 experiments. Means and 95% confidence intervals are indicated. The statistical significance of genotypic effects on fitness and its components in individual years was tested with contrasts. * $P < 0.05$, ** $P < 0.01$, *** $P < 0.001$.

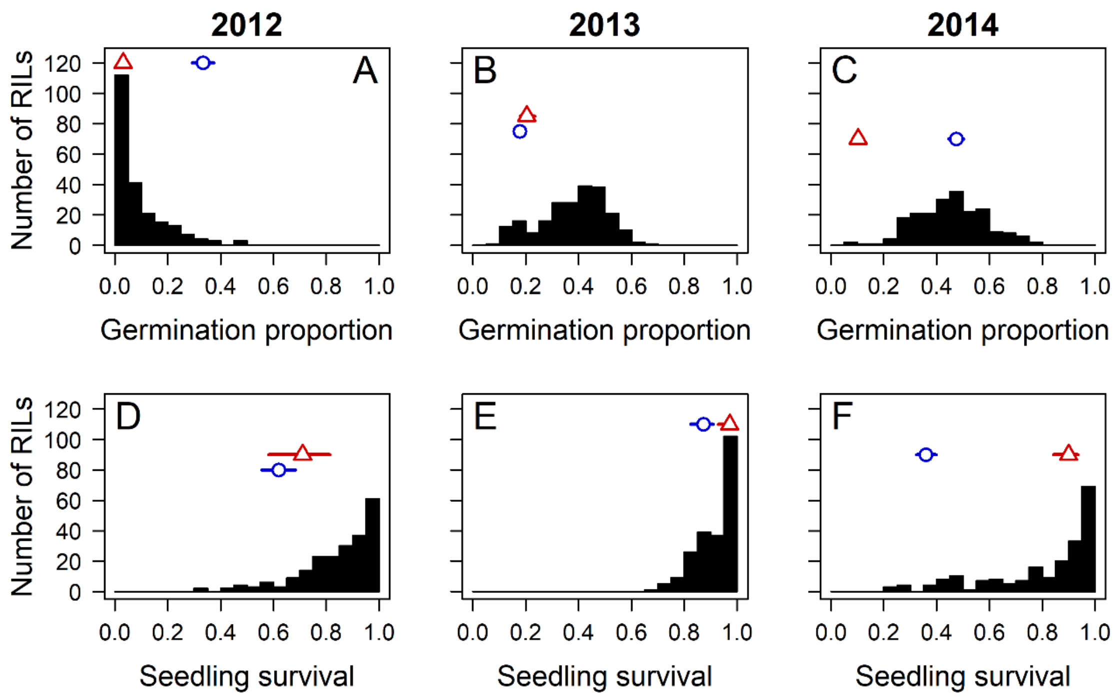

Fig. S3. Frequency distributions of mean proportion of viable seeds germinating (A, B, C) and mean seedling survival (D, E, F) in the 2012 (219 RILs), 2013 (220 RILs) and 2014 (204 RILs) experiments conducted at the native site of the Swedish genotype. Mean values with corresponding 95% confidence intervals of the Italian (red triangle) and Swedish (blue circle) parental genotypes are indicated for comparison. RILs and parental genotypes were planted as primary dormant seeds soon after maturation at the experimental site.

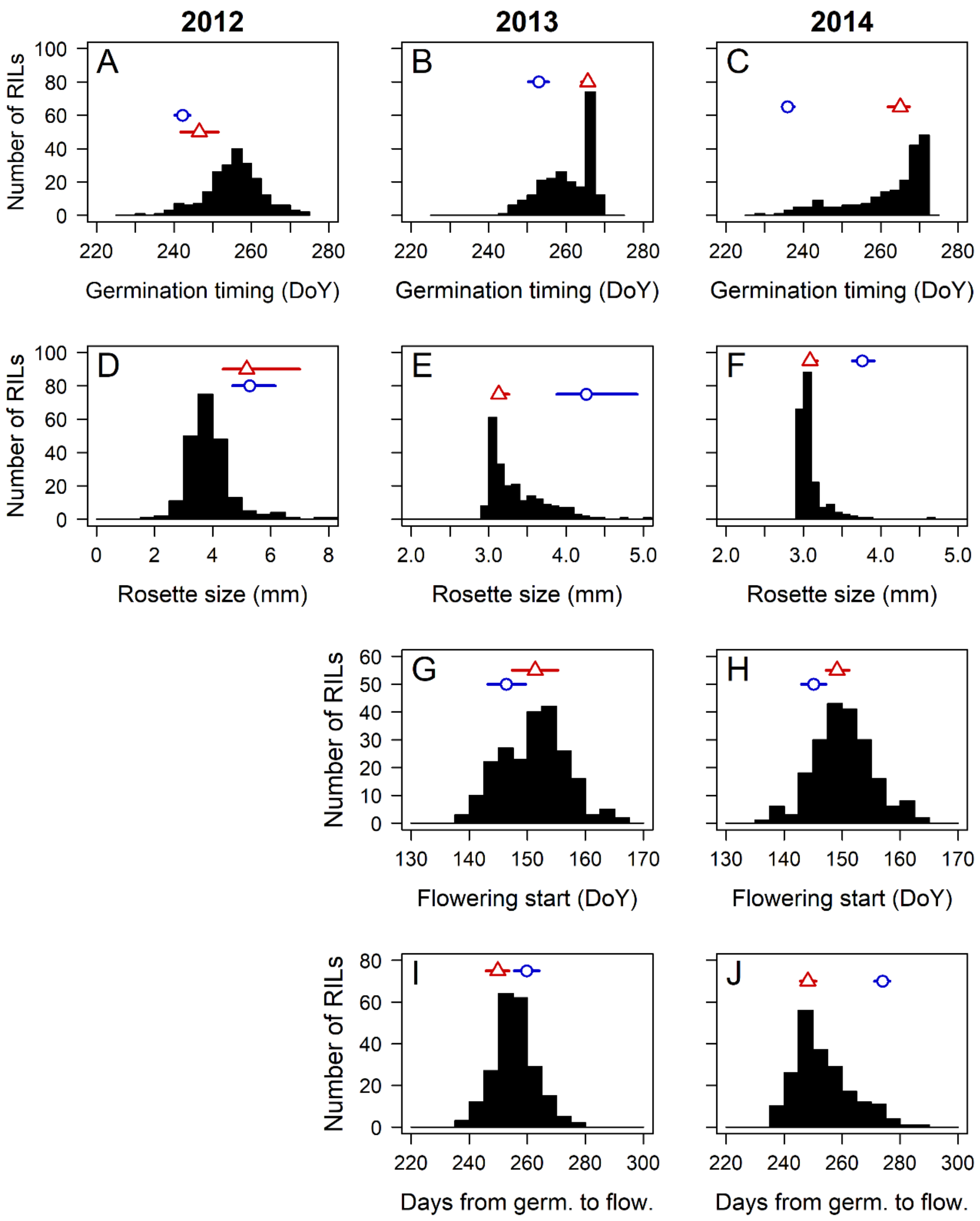

Fig. S4. Frequency distributions of mean timing of germination (day of year; A, B, C), rosette size (diameter in mm; D, E, F), flowering start (day of year; G, H), and number of days from germination until flowering start (I, J) in the 2012 (219 RILs), 2013 (220 RILs) and 2014 (204 RILs) experiments conducted at the native site of the Swedish genotype. The mean values with corresponding 95% confidence intervals of the Italian (red triangle) and Swedish (blue circle) parental genotypes are also indicated. Note the difference in the scale of the x-axis for rosette size in (D) compared to (E) and (F). RILs and parental genotypes were planted as primary dormant seeds soon after maturation at the experimental site.

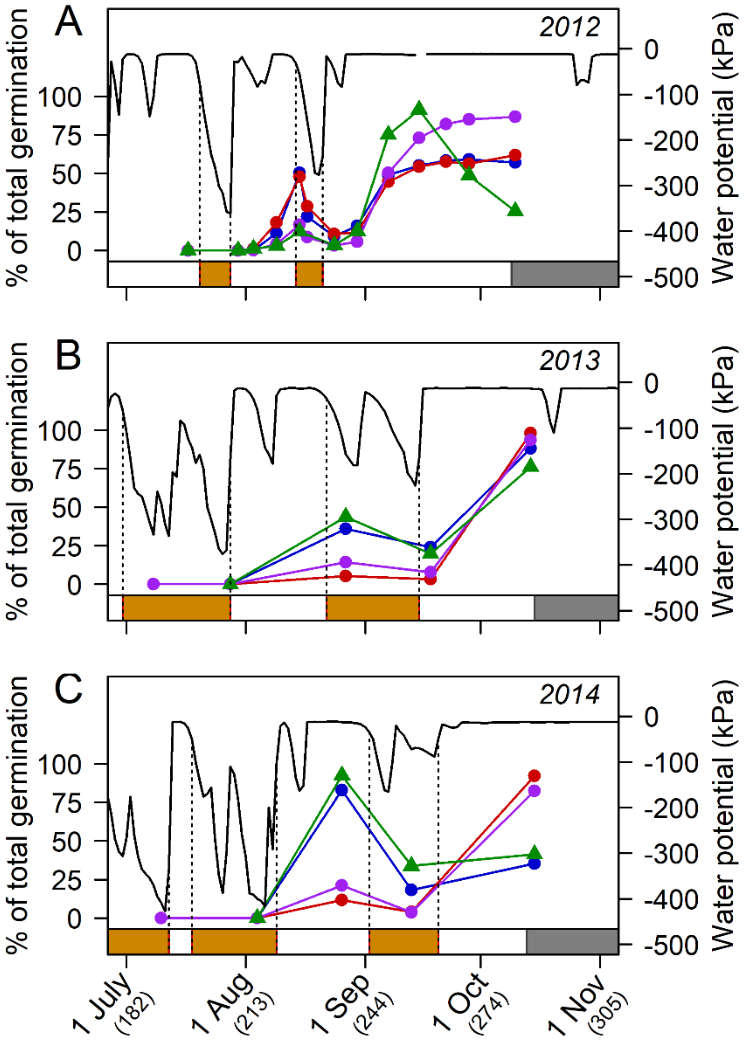

Fig. S5. Mean number of living seedlings expressed as a percentage of total germination of the Italian genotype (red), the Swedish genotype (blue) and the RIL population (purple) planted as primary dormant seeds shortly after maturation at the field site in (A) the 2012, (B) the 2013, and (C) the 2014 experiments. For comparison, the same measure obtained in 25 plots in the local natural population (green) is given. Soil water potential recorded at the experimental site during the germination period in 2012, 2013, and 2014 is shown in black. Unfavourable germination conditions, consisting of dry periods (orange, soil water potential <-30 kPa for more than seven days) and the onset of winter (grey, mean daily soil temperature below 5 °C for more than two subsequent days, see Fig. 5), are indicated on the x-axis. Date (day of year) is given below the x-axis.

Table S1. Fitness and fitness components (mean ± SE) of the Italian and Swedish genotypes planted as primary dormant (D) and after-ripened non-dormant (ND) seeds at the experimental site in 2012, 2013 and 2014. Primary dormant seeds were sown soon after seeds had matured at the field site, the after-ripened non-dormant seeds were sown during the natural germination period of the local *A. thaliana* population. Total fitness (number of fruits produced per viable seed planted), and its components germination proportion (proportion of viable seeds that germinated), seedling survival (proportion of seedlings surviving from germination until the end of the germination period just before winter arrival), adult survival (proportion of plants surviving from the end of the germination period until fruiting) and fecundity (number of fruits produced per surviving plant). The selection coefficients (*s*) quantify the strength of selection (*s* = 1 - [mean fitness of less fit genotype / mean fitness of fittest genotype]) against the non-local Italian genotype, and was calculated based on total fitness as well as its components. Selection against the local genotype is indicated with a negative selection coefficient. The 95% confidence intervals of s were obtained by bootstrapping. Significant selection coefficients (95% confidence intervals not overlapping zero) are shown in bold. In addition, the grand means ± SE for the Recombinant Inbred Lines (based on RIL mean trait values), the number of RILs, and the mean number of replicates per RIL are given.

Variable	Year	Dormancy	Italian genotype	*n*	Swedish genotype	*n*	*s* (95% CI)	RIL population	No. RILs	Mean *n* per RIL
Total fitness	2012	D	0.105 ± 0.034	79	1.365 ± 0.233	80	**0.92** (0.83, 0.96)	0.516 ± 0.070	219	9.7
		ND	0.267 ± 0.091	80	3.596 ± 0.637	79	**0.93** (0.83, 0.96)	-	-	-
	2013	D	0.353 ± 0.126	72	2.301 ± 0.626	72	**0.85** (0.69, 0.93)	1.668 ± 0.248	220	9.0
		ND	0.126 ± 0.037	54	0.241 ± 0.073	54	0.48 (-0.01, 0.76)	-	-	-
	2014	D	0.233 ± 0.028	80	0.754 ± 0.081	80	**0.69** (0.57, 0.77)	0.820 ± 0.031	204	5.0
		ND	1.028 ± 0.239	30	1.307 ± 0.223	30	0.21 (-0.42, 0.54)	-	-	-
Germ. proportion	2012	D	0.031 ± 0.004	79	0.333 ± 0.021	80	**0.91** (0.87, 0.93)	0.090 ± 0.007	219	9.7
		ND	0.282 ± 0.027	80	0.835 ± 0.011	79	**0.66** (0.60, 0.73)	-	-	-
	2013	D	0.204 ± 0.014	80	0.177 ± 0.011	80	-0.13 (-0.26, 0.03)	0.380 ± 0.008	220	10.0
		ND	0.340 ± 0.013	60	0.244 ± 0.015	60	**-0.28** (-0.38, -0.17)	-	-	-
	2014	D	0.103 ± 0.007	80	0.475 ± 0.014	80	**0.78** (0.75, 0.82)	0.459 ± 0.009	204	5.0
		ND	0.805 ± 0.031	30	0.467 ± 0.039	30	**-0.42** (-0.52, -0.31)	-	-	-
Seedling survival	2012	D	0.71 ± 0.06	46	0.62 ± 0.03	80	-0.13 (-0.25, 0.05)	0.85 ± 0.01	217	7.0
		ND	0.84 ± 0.02	67	0.88 ± 0.01	79	0.04 (-0.01, 0.11)	-	-	-
	2013	D	0.97 ± 0.01	80	0.87 ± 0.02	79	**-0.10** (-0.15, -0.06)	0.92 ± 0.00	220	10.0
		ND	1.00 ± 0.00	60	1.00 ± 0.00	60	0	-	-	-
	2014	D	0.90 ± 0.02	77	0.36 ± 0.02	80	**-0.60** (-0.64, -0.55)	0.81 ± 0.01	204	5.0
		ND	1.00 ± 0.00	30	1.00 ± 0.00	30	0	-	-	-
Adult survival	2012	D	0.75 ± 0.07	40	0.90 ± 0.03	79	**0.17** (0.001, 0.34)	0.78 ± 0.01	217	6.7
		ND	0.45 ± 0.06	66	0.91 ± 0.03	79	**0.50** (0.36, 0.64)	-	-	-
	2013	D	0.30 ± 0.04	72	0.47 ± 0.04	72	**0.36** (0.13, 0.55)	0.34 ± 0.01	220	9.0
		ND	0.13 ± 0.03	54	0.32 ± 0.05	54	**0.60** (0.28, 0.79)	-	-	-

	2014	D	0.61 ± 0.04	76	0.75 ± 0.04	80	**0.19** (0.06, 0.31)	0.68 ± 0.01	204	5.0
		ND	0.45 ± 0.07	30	0.73 ± 0.05	30	**0.39** (0.13, 0.59)	-	-	-
Fecundity	2012	D	7.55 ± 1.93	33	7.96 ± 1.05	71	0.05 (-0.68, 0.45)	6.28 ± 0.42	215	5.4
		ND	2.53 ± 0.66	32	5.68 ± 1.02	72	**0.55** (0.13, 0.75)	-	-	-
	2013	D	8.62 ± 3.95	37	23.96 ± 4.82	52	**0.64** (0.05, 0.86)	17.25 ± 2.15	220	5.0
		ND	4.56 ± 2.27	16	2.86 ± 0.53	29	-0.37 (-0.81, 0.44)	-	-	-
	2014	D	4.48 ± 0.33	63	5.72 ± 0.44	72	**0.22** (0.04, 0.38)	3.45 ± 0.11	204	4.2
		ND	2.75 ± 0.43	20	4.19 ± 0.64	27	**0.34** (0.05, 0.56)	-	-	-

Table S2. The effects of year (2012, 2013 vs. 2014 experiment), genotype (Italian vs. Swedish) and dormancy category (primary dormant seeds planted during the natural seed dispersal period vs. after-ripened non-dormant seeds planted during the natural germination period of the local population) on total fitness and its components germination proportion, seedling survival, adult survival and fecundity. The significance of each explanatory variable and interaction term was tested with GLM by comparing the change in deviance between the full model and a reduced model.

Response	Source of variation	df/Δdf	Deviance	F or χ value	*P*
Total fitness[c]	**Genotype**	789/1	514.0	105.8	**< 0.001**
	**Dormancy**	789/1	415.7	7.4	**0.006**
	Year	789/2	411.5	3.3	0.195
	**Genotype × dormancy**	785/1	408.3	5.2	**0.022**
	**Genotype × year**	785/2	418.4	15.3	**< 0.001**
	**Dormancy × year**	785/2	487.2	84.1	**< 0.001**
	Genotype × dormancy × year	780/2	405.9	5.9	0.051
Germ. prop.[a]	**Genotype**	817/1	216.0	184.3	**< 0.001**
	**Dormancy**	817/1	244.6	316.7	**< 0.001**
	**Year**	817/2	200.8	57.2	**< 0.001**
	**Genotype × dormancy**	813/1	128.7	95.2	**< 0.001**
	**Genotype × year**	813/2	162.2	224.7	**< 0.001**
	**Dormancy × year**	813/2	116.6	96.0	**< 0.001**
	**Genotype × dormancy × year**	808/2	104.2	70.1	**< 0.001**
Seedling surv.[a]	**Genotype**	767/1	249.1	105.9	**< 0.001**
	**Dormancy**	767/1	276.0	200.0	**< 0.001**
	**Year**	767/2	282.4	111.1	**< 0.001**
	**Genotype × dormancy**	763/1	172.4	5.5	**0.019**
	**Genotype × year**	763/2	187.3	35.8	**< 0.001**
	**Dormancy × year**	763/2	189.0	39.7	**< 0.001**
	Genotype × dormancy × year	758/2	171.1	0	1
Adult survival[b]	**Genotype**	731/1	610.5	47.4	**< 0.001**
	**Dormancy**	731/1	577.9	14.8	**< 0.001**
	**Year**	731/2	685.7	122.6	**< 0.001**
	**Genotype × dormancy**	727/1	553.8	4.1	**0.042**
	Genotype × year	727/2	554.2	4.5	0.11
	Dormancy × year	727/2	551.2	1.5	0.47
	Genotype × dormancy × year	722/2	549.7	1.3	0.52
Fecundity[c]	**Genotype**	523/1	598.6	19.0	**< 0.001**
	**Dormancy**	523/1	625.2	45.6	**< 0.001**
	**Year**	523/2	626.2	46.6	**< 0.001**
	Genotype × dormancy	519/1	578.8	0.0	0.86
	Genotype × year	519/2	579.9	1.1	0.57
	**Dormancy × year**	519/2	596.1	17.3	**< 0.001**
	**Genotype × dormancy × year**	514/2	595.0	18.5	**< 0.001**

[a]Quasibinomial error distribution with logit link, F-test
[b]Binomial error distribution with logit link, chi-square test
[c]Negative binomial error distribution with log link, chi-square test

Table S3. Life-history traits of the Italian and Swedish genotypes planted as primary dormant seeds (D) during the natural seed dispersal period, and as after-ripened non-dormant seeds (ND) during the natural germination period of the local population at the field site in Sweden in 2012, 2013 and 2014. Means ± SE are given for germination time (day of year), rosette size (diameter in mm, at the end of the germination period), flowering start (day of year) and the number of days from germination until flowering start. Flowering start was not estimated in the 2012 experiment. In addition, the corresponding grand means ± SE of the Recombinant Inbred Lines (based on RIL mean trait values), the number of RILs, and the mean number of replicates per RIL are given.

**Variable**	**Year**	**Dormancy category**	**Italian genotype**	***n***	**Swedish genotype**	***n***	**RIL population**	**No. RILs**	**Mean *n* per RIL**
Germ. timing (DoY)	2012	D	246.5 ± 2.5	46	242.2 ± 1.0	80	255.6 ± 0.5	217	7.0
		ND	255.0 ± 0.8	67	252.1 ± 0.1	79	-	-	-
	2013	D	265.6 ± 0.6	80	252.9 ± 1.3	79	260.6 ± 0.4	220	10.0
		ND	267.1 ± 0.3	60	266.6 ± 0.5	60	-	-	-
	2014	D	265.1 ± 1.4	77	235.9 ± 0.8	80	261.8 ± 0.7	204	5.0
		ND	271.7 ± 0.2	30	268.7 ± 0.8	30	-	-	-
Rosette size (mm)	2012	D	5.2 ± 0.60	43	5.3 ± 0.37	80	3.97 ± 0.08	217	6.7
		ND	3.6 ± 0.15	72	3.5 ± 0.08	79	-	-	-
	2013	D	3.1 ± 0.05	80	4.3 ± 0.25	78	3.37 ± 0.03	220	10.0
		ND	3.1 ± 0.02	60	3.1 ± 0.03	60	-	-	-
	2014	D	3.1 ± 0.03	75	3.8 ± 0.07	80	3.09 ± 0.01	204	5.0
		ND	3.0 ± 0.00	30	3.0 ± 0.04	30	-	-	-
Flowering start (DoY)	2012	D	*NA*	*NA*	*NA*	*NA*	*NA*	*NA*	*NA*
		ND	*NA*	*NA*	*NA*	*NA*	-	-	-
	2013	D	151.3 ± 2.0	32	146.4 ± 1.7	34	151.3 ± 0.4	219	4.0
		ND	151.4 ± 3.3	14	150.4 ± 1.7	28	-	-	-
	2014	D	149.1 ± 1.0	64	145.1 ± 1.1	75	150.3 ± 0.4	204	4.2
		ND	148.7 ± 2.3	22	152.3 ± 2.0	26	-	-	-
Number of days from germination to flowering	2012	D	*NA*	*NA*	*NA*	*NA*	*NA*	*NA*	*NA*
		ND	*NA*	*NA*	*NA*	*NA*	-	-	-
	2013	D	249.8 ± 2.0	32	259.8 ± 2.2	34	255.6 ± 0.5	219	4.0
		ND	249.8 ± 3.6	14	248.4 ± 1.8	28	-	-	-
	2014	D	248.2 ± 1.4	64	273.9 ± 1.3	75	253.5 ± 0.7	204	4.2
		ND	241.9 ± 2.3	22	248.3 ± 2.1	26	-	-	-

Table S4. The effects of year (2012, 2013 vs. 2014 experiment), genotype (Italian vs. Swedish) and dormancy category (primary dormant seeds planted during the natural seed dispersal period vs. after-ripened non-dormant seeds planted during the natural germination period of the local population) on germination timing (day of year), rosette size (diameter in mm), flowering start (day of year) and number of days from germination to flowering start. The significance of each explanatory variable and interaction term was tested with GLM by comparing the change in deviance between the full model and a reduced model.

Response	Source of variation	df/Δdf	Deviance	F or χ value	P
Germ. timing[a]	**Genotype**	767/1	397.5	79.4	**< 0.001**
	**Dormancy**	767/1	403.1	85.0	**< 0.001**
	**Year**	767/2	424.1	106.0	**< 0.001**
	**Genotype × dormancy**	763/1	257.4	26.5	**< 0.001**
	**Genotype × year**	763/2	259.9	29.1	**< 0.001**
	**Dormancy × year**	763/2	249.2	18.4	**< 0.001**
	**Genotype × dormancy × year**	758/2	230.9	16.5	**< 0.001**
Rosette size[b]	**Genotype**	766/1	356.9	19.0	**< 0.001**
	**Dormancy**	766/1	392.4	96.5	**< 0.001**
	**Year**	766/2	400.0	56.6	**< 0.001**
	**Genotype × dormancy**	762/1	335.0	9.5	**0.002**
	**Genotype × year**	762/2	334.7	4.5	**0.011**
	**Dormancy × year**	762/2	339.4	9.8	**< 0.001**
	Genotype × dormancy × year	757/2	330.8	2.5	0.08
Flowering start[a]	Genotype	294/1	191.5	3.5	0.063
	**Dormancy**	294/1	192.0	4.0	**0.045**
	Year	294/1	188.8	0.8	0.38
	Genotype × dormancy	291/1	187.6	3.8	0.050
	Genotype × year	291/1	184.2	0.5	0.49
	Dormancy × year	291/1	184.0	0.3	0.60
	Genotype × dormancy × year	288/1	183.7	0.3	0.56
Number of days from germination to flowering[a]	**Genotype**	293/1	245.3	68.2	**< 0.001**
	**Dormancy**	293/1	218.2	41.2	**< 0.001**
	**Year**	293/1	181.2	4.2	**0.041**
	**Genotype × dormancy**	290/1	159.1	13.4	**< 0.001**
	**Genotype × year**	290/1	156.4	10.7	**0.001**
	**Dormancy × year**	290/1	152.3	6.6	**0.010**
	Genotype × dormancy × year	287/1	145.7	0.7	0.39

[a]Poisson error distribution with log link, chi-square test
[b]Quasipoisson error distribution with log link, F-test

Table S5. The effects of germination timing (day of year) and germination proportion on rosette size (diameter in mm) analysed separately by year (2012, 2013, and 2014 experiment) and dormancy category (D, primary dormant seeds planted during the seed dispersal period of the local population; ND, after-ripened non-dormant seeds planted during the germination period of the local population). The significance of each explanatory variable was tested with a generalized linear model with quasipoisson error distribution and log link by comparing the change in deviance between the full model and a reduced model with an F-test.

Year	Dormancy	Source of variation	df/Δdf	Estimate ± SE	Deviance	F value	P
2012	D	**Germtime**	121/1	**-0.025 ± 0.005**	199.9	36.4	**< 0.001**
		Germprop	121/1	-0.062 ± 0.252	153.2	0.07	0.78
	ND	**Germtime**	144/1	**-0.020 ± 0.004**	35.0	23.4	**< 0.001**
		Germprop	144/1	-0.135 ± 0.072	30.8	3.7	0.06
2013	D	**Germtime**	157/1	**-0.020 ± 0.002**	82.2	97.8	**< 0.001**
		**Germprop**	157/1	**0.541 ± 0.236**	52.4	6.1	**0.01**
	ND	**Germtime**	119/1	**-0.014 ± 0.002**	1.8	76.0	**< 0.001**
		Germprop	119/1	0.022 ± 0.043	1.1	0.3	0.61
2014	D	**Germtime**	154/1	**-0.008 ± 0.001**	11.7	90.5	**< 0.001**
		Germprop	154/1	-0.078 ± 0.067	7.4	1.3	0.25
	ND	**Germtime**	59/1	**-0.007 ± 0.002**	0.5	13.4	**< 0.001**
		Germprop	59/1	0.024 ± 0.028	0.4	0.8	0.38

Table S6. Summary statistics of the sequential model evaluation in the path analysis of RIL mean values in the 2012, 2013 and 2014 experiments. The RILs were planted as primary dormant seeds during the natural seed dispersal period. The χ2 goodness-of-fit statistics are given, and the Approximate Fit Indices Comparative Fit Index (CFI), Tucker-Lewis Index (TLI), Root Mean Squared Error (RMSEA) with 90% confidence interval and corresponding *P*-value, and the standardized maximum normalized residual (SRMR). First, paths were sequentially added to the initial model based on the modification index (MI > 8.8), and subsequently one by one removed based on the Z-value ($P > 0.05$). Paths are presented in the "lavaan" syntax: A ~ B indicates B regressed onto A, A ~~ B indicates the covariance of A and B (Rosseel, 2012). The final models are indicated with an asterisk.

	**χ2 goodness-of-fit statistics**				**Approximate Fit Indices**				
Path addition (+) or deletion (-)	*n*	χ^2_M	df_M	P_M	CFI	TLI	RMSEA (90% CI)	$P_{RMSEA} < 0.05$	SRMR
**2012 experiment**									
Initial model *	214	9.6	9	0.383	0.998	0.996	0.018 (0.000, 0.078)	0.747	0.049
**2013 experiment**									
Initial model	217	83.4	13	0.000	0.905	0.796	0.158 (0.129, 0.188)	0.000	0.105
+ germprop ~~ germtime	217	47.4	12	0.000	0.952	0.887	0.117 (0.087, 0.148)	0.000	0.041
+ seedlsurv ~~ size	217	24.7	11	0.010	0.981	0.952	0.076 (0.039, 0.112)	0.111	0.036
+ size ~ dormancy	217	17.3	10	0.067	0.990	0.972	0.058 (0.000 0.099)	0.335	0.032
- flowtime ~ dormancy	217	17.7	11	0.089	0.991	0.977	0.053 (0.000, 0.093)	0.410	0.033
- fecund ~ flowstart*	217	8.4	8	0.392	0.999	0.998	0.016 (0.000, 0.078)	0.750	0.024
**2014 experiment**									
Initial model	203	78.2	13	0.000	0.943	0.878	0.157 (0.126, 0.190)	0.000	0.072
+ seedlsurv ~~ size	203	38.6	12	0.000	0.979	0.950	0.105 (0.069, 0.142)	0.008	0.075
+ fecund ~ germtime	203	24.6	11	0.010	0.989	0.972	0.078 (0.036, 0.120)	0.119	0.055
+ germprop ~~ germtime	203	14.7	10	0.144	0.996	0.989	0.048 (0.000, 0.096)	0.473	0.030
- flowtstart ~ dormancy	203	15.2	11	0.175	0.997	0.991	0.043 (0.000, 0.090)	0.539	0.034
- fecund ~ size*	203	17.1	12	0.146	0.996	0.990	0.046 (0.000, 0.091)	0.509	0.035

Table S7. Unstandardized path coefficients ± SE and corresponding *P*-values in the final models of the path analysis for the 2012, 2013 and 2014 experiments. Dormancy, germination proportion, seedling survival and adult survival are quantified as proportions, germination timing and flowering time in days, rosette size as the diameter in mm and fecundity as number of fruits. The variables that were rescaled prior to analysis (germination timing, flowering time and fecundity) are back-transformed to their original units. Flowering time was not recorded in the 2012 experiment.

Path		2012		2013		2014	
Independent variable	Dependent variable	Estimate ± SE	*P*	Estimate ± SE	*P*	Estimate ± SE	*P*
Dormancy	Germ. proportion	-0.186 ± 0.019	< 0.001	0.142 ± 0.025	< 0.001	0.073 ± 0.025	0.004
Germ. timing	Seedling survival	0.015 ± 0.001	< 0.001	0.011 ± 0.000	< 0.001	0.020 ± 0.000	< 0.001
Rosette size	Adult survival	0.047 ± 0.012	< 0.001	0.133 ± 0.018	< 0.001	0.122 ± 0.042	0.004
Rosette size	Fecund	2.5 ± 1.0	0.015	36.7 ± 9.2	< 0.001	–	–
Flowering start	Fecund	NA	NA	–	–	-0.154 ± 0.013	< 0.001
Germ. timing	Fecund	–	–	–	–	-0.050 ± 0.009	< 0.001
Dormancy	Germ. timing	8.4 ± 1.3	< 0.001	10.5 ± 1.0	< 0.001	21.1 ± 1.9	< 0.001
Germ. timing	Rosette size	-0.071 ± 0.012	< 0.001	-0.045 ± 0.003	< 0.001	-0.013 ± 0.002	< 0.001
Dormancy	Rosette size	–	–	-0.151 ± 0.060	0.012	–	–
Rosette size	Flowering start	NA	NA	-4.3 ± 0.9	< 0.001	-10.3 ± 3.0	0.001
